# A randomized waitlist-controlled trial comparing detached mindfulness and cognitive restructuring in obsessive-compulsive disorder

**DOI:** 10.1371/journal.pone.0213895

**Published:** 2019-03-20

**Authors:** Christian Rupp, Charlotte Jürgens, Philipp Doebler, Fabian Andor, Ulrike Buhlmann

**Affiliations:** 1 Institute of Psychology, Westfälische Wilhelms-University Münster, Fliednerstrasse 21, Münster, Germany; 2 Christoph-Dornier-Stiftung, Schorlemerstrasse 26, Münster, Germany; 3 Department of Statistics, TU Dortmund University, Dortmund, Germany; University of California Los Angeles, UNITED STATES

## Abstract

**Objective:**

Whereas research has demonstrated the efficacy of cognitive restructuring (CR) for obsessive-compulsive disorder (OCD), little is known about the efficacy of specific metacognitive interventions such as detached mindfulness (DM). Therefore, this study compared the efficacy of CR and DM as stand-alone interventions.

**Design:**

We conducted a randomized waitlist-controlled trial. *n* = 43 participants were randomly assigned to either DM or CR. Out of those participants, *n* = 21 participants had been previously assigned to a two-week waitlist condition.

**Materials and methods:**

In both conditions, treatment comprised four double sessions within two weeks. Assessment took place at baseline (Pre1), after treatment (Post) and four weeks after the end of treatment (FU). There was a second baseline assessment (Pre2) in the waitlist group. Independent evaluators were blinded concerning the active condition. Adherence and competence ratings for the two therapists were obtained from an independent rater.

**Results:**

40 patients completed the treatment. Two patients dropped out because of exacerbated depression. There were no further adverse events. Both CR and DM were shown to be superior to waitlist and equally effective at reducing OCD symptoms from pre to post assessment as measured with the Y-BOCS (CR: *d* = 1.67, DM: *d* = 1.55). In each of the two treatment conditions, eight patients (40%) exhibited a clinical significant change at post assessment.

**Conclusions:**

The results of this clinical trial suggest the potential efficacy of DM as a stand-alone intervention for OCD, however, our findings need to be interpreted with caution. Results indicate that both CR and DM should be considered as possible alternative treatments for OCD, whereas the working mechanisms of DM have yet to be elucidated.

## Introduction

According to DSM-5, obsessive-compulsive disorder (OCD) is defined by intrusive and unwanted thoughts, images or urges (from now on referred to as obsessive thoughts or obsessions) and/or repetitive overt behaviors or mental acts used to reduce fear or distress caused by the above-named intrusive mental event [1]. Concerning treatment guidelines [2], cognitive behavioral therapy comprising exposure and response prevention (ERP), i. e., confronting patients with triggering stimuli while encouraging them to refrain from compulsions, as well as psychopharmacological treatment with selective serotonin reuptake inhibitors are considered as gold standard in treating OCD. However, around 30% of the patients treated with cognitive behavioral therapy either do not respond to this treatment [3] or decline the stressful and demanding exposure interventions. Moreover, around 20% of patients drop out of therapy [4], so that it seems crucial to further improve current treatments and to promote the development of alternative treatments.

### Metacognitive therapy

Wells and Matthews [5] developed the Self-Regulatory Executive Function (S-REF) model of psychological disorders, which accentuates the role of metacognition in psychological disorders. Regarding OCD, the metacognitive model [6] emphasizes the meaning of dysfunctional metacognitive beliefs in the development and maintenance of OCD. Most importantly, it assumes that obsessive thoughts activate metacognitive beliefs concerning the meaning of thoughts, such as the assumption that thoughts represent or have an impact on reality (referred to as Thought-Action-Fusion, Thought-Object-Fusion and Thought-Event-Fusion, respectively). Based on these fusion-beliefs, obsessive thoughts are perceived as threatening, thus activating negative emotions (e.g., fear, guilt, distress) as well as metacognitive thoughts about the need to perform rituals or to engage in thought suppression. These rituals then serve to reduce the perceived threat, while internal metacognitive criteria about stop signals serve as reference when to end the rituals.

Metacognitive therapy (MCT) of OCD based on the model mentioned above focuses on challenging metacognitive beliefs, whereas it explicitly does not include questioning of thought *content*. Instead, MCT comprises techniques such as *detached mindfulness* (DM), *exposure and response commission* and Socratic questioning of metacognitive beliefs. According to Wells [6], DM is a key technique in treating OCD as it offers an overall new approach to dealing with intrusive thoughts. Wells and Matthews [5] described DM as a technique to develop meta-consciousness, i. e., a state of mind in which the self and cognitive events are separated from one another. While in this state, the person is aware of his or her thoughts only being mental events–which s/he learns to solely observe in a passive way.

Efficacy of complex MCT treatment programs for OCD has been proven in a number of trials with, however, comparably small sample sizes [7–10]. However, only a few studies have examined the efficacy of single treatment components of MCT.

Wahl et al. [11] compared the efficacy of a mindfulness-based strategy with a distraction strategy during brief exposure to obsessive thoughts in a sample of 30 OCD patients. Significant decreases in anxiety and urge to neutralize between time of experimental manipulation and to post assessment were only found in the mindfulness-based strategy group. Firouzabadi and Shareh [12] examined the efficacy of DM in a single case study treating an OCD patient. The treatment led to a 26-point-decrease in Y-BOCS score from pre- to follow-up-assessment. Ludvik and Boschen [13] compared the efficacy of DM, cognitive restructuring (CR) and a control task (reading an unrelated scientific article) in reducing experimentally induced memory distrust and urge to check in a sample of 65 undergraduate students. Compared with the control task, participants in the control group were significantly more likely to check than participants receiving DM or CR, whereas only DM led to a significant improvement in memory confidence at post-test. However, the experimental manipulation in this study cannot be compared to an actual treatment since it was delivered in the form of a written instruction and was limited to one single occasion.

As MCT focuses on changing one’s relationship to one’s own thoughts and since DM embodies a direct way of training this new way of dealing with mental events such as intrusive thoughts, it is expected to lead to a reduction in OCD symptoms according to the metacognitive model. Thus, it seems essential to examine the efficacy of DM as a stand-alone intervention in the treatment of OCD.

### Cognitive restructuring

In contrast to MCT, the cognitive models of OCD proposed by Salkovskis [14, 15] and Rachman [16] emphasize the impact of distorted cognitions in the development and maintenance of OCD. Both authors suggest that not the intrusions themselves but rather the meaning attached to them (referred to as “automatic thoughts” by Salkovskis and as “misinterpretations” by Rachman) are responsible for negative emotional consequences—such as when interpreting having the intrusion as proof for being a “bad person”. Evidence for this assumption comes, for example, from studies in which non-clinical samples were shown to report intrusions without being distressed [17, 18]. Salkovskis [14] suggested that these automatic thoughts are caused by specific individual beliefs about being personally responsible for harm, beliefs that having thoughts about an action is the same as accomplishing an action as well as beliefs about the need to control thoughts. Similar distortions have been described by the Obsessive Compulsive Cognitions Working Group (OCCWG) [19] who, in addition to Salkovskis, also list perfectionism, intolerance for uncertainty and overestimation of threat as relevant cognitive distortions in OCD.

Built on the cognitive model, which emphasizes that dysfunctional cognitions in response to intrusions constitute the maintaining factor in OCD, cognitive restructuring (CR) of OCD targets distorted cognitions/appraisals of obsessive thoughts primarily by using Socratic questioning. Thus, unlike DM, which teaches patients to *passively* observe and disassociate themselves from their intrusions while refraining from any sort of conceptual processing, CR provides patients with an *active* strategy of dealing with obsessive thoughts by questioning the appraisals attached to them. As such, DM and CR can be regarded as two entirely different approaches to the same problem.

Efficacy of CR in the treatment of OCD has been shown in a number of trials, of which, however, the majority comprised behavioral experiments, which can be considered as having some overlap with exposure tasks because they involve confronting patients with triggering stimuli (such as locking the door without checking if it is locked in checking-related OCD) while encouraging them to refrain from compulsions. Whereas ERP would traditionally highlight that over time, feelings of fear, disgust and tension decrease (which is often referred to as *habituation*), behavioral experiments within a CR approach would emphasize that a certain cognition has been contradicted (e. g., because the expected burglary did not occur).

The studies by Wilhelm et al. [20, 21] and Belloch et al. [22] all demonstrate the efficacy of complex cognitive treatment programs including behavioral experiments over several weeks. According to meta-analyses [4, 23, 24], CR proves to be an effective method for treating OCD—for lack of any significant differences concerning treatment efficacy of CR vs. ERP. Various other studies have investigated the efficacy of CR as compared to ERP, most of which showed no considerable differences concerning efficacy [25, 26], with one study suggesting ERP to be superior to CR in terms of recovery rates [27] and one showing the opposite result [28]. It should be noted in this context that the only one of the above-named studies whose protocol did not include behavioral experiments was the one effectiveness study by Belloch et al. [29], so that there is little evidence for the efficacy of “purely Beckian” cognitive restructuring excluding this element. Finally, a number of meta-analyses [4, 23, 24], arrive at the conclusion that CR proves to be an effective method for treating OCD.

Given the two seemingly contradictory strategies for treating OCD, the main goal of our study was to examine the efficacy of 1) teaching patients a passively observing relation to one’s own thoughts (i. e., DM) on the one hand and 2) leading them to actively question distorted appraisals and beliefs (i. e., CR) on the other hand. Since the efficacy of CR for OCD, often being compared with ERP, has been widely demonstrated (with most protocols, however, involving behavioral experiments), we regarded CR as the more established treatment approach that we decided to compare with DM, which represents a more recent and conceptually fresh take on treating OCD. In either case, our interest was to elucidate the efficacy of both approaches as stand-alone interventions, which is why we compared a purely “Beckian” form of CR with the specific intervention of DM.

## Materials and methods

### Study design

The study protocol for this clinical trial was registered at ClinicalTrials.gov under the ID NCT03002753 and the title “Dealing With Intrusive Thoughts in OCD—a Comparison of Detached Mindfulness and Cognitive Restructuring”(Protocol ID: CDS-MS-JR-2016, URL: https://clinicaltrials.gov/ct2/results?cond=&term=NCT03002753&cntry=&state=&city=&dist=). The design of the study can be best described as a randomized delayed-intervention controlled trial with an underlying parallel design concerning the two active conditions (CR/DM). Participants randomized to the non-waitlist (NWL) group started their treatment at the beginning of the week following initial assessment (Pre1) whereas participants randomized to the waitlist control group (WL) started treatment with a delay of two weeks.

The study protocol was reviewed and approved of by the ethics committee of the Department of Psychology and Sport Science at the University of Münster, Germany (approval number: 2016-37-UB). All participants provided written informed consent after the study procedure had been fully explained. The study was conducted between January 2017 (start of data collection) and July 2018 (end of data collection), whereas recruitment began in December 2016 and was completed in June 2017. The last follow-up assessment marking the end of the active phase of the study was on July 12, 2018.

### Power analysis

Sample size was estimated on the basis of an a priori conducted power analysis. Importantly, we were not interested in finding differences between the two treatment conditions concerning efficacy. Thus, power analysis focused on the sample size required to find pre to post treatment effects in each of the two treatment groups. Based on the literature reviewed above it was difficult to estimate the expected effect size *f* for a short-term but intense stand-alone intervention of DM or CT in a clinical sample. We arrived at an estimate for *f* ranging from 0.25 to 0.40 for a between-within-interaction in a 2x2 repeated measures ANOVA (which corresponds to a Cohen’s *d* of 0.5 and 0.8, respectively). Given an *α* level of 0.05, a power of *β* = 0.90 and a correlation between the two assessment points (labeled T1 and T2, respectively) of *r* = 0.5, the corresponding total sample size ranged from 46 to 20 participants. We originally planned to recruit a total of 60 participants, reduced the targeted sample size to a total of 40 participants in March 2017 due to recruitment difficulties.

### Procedure

Participants were recruited via postings in social media including German OCD awareness online platforms as well as via posters in university buildings and flyers distributed in surrounding psychiatric, neurologic and dermatologic practices and outpatient departments as well as in local physicians’ practices and pharmacies. Recruitment also involved repeated advertisements in local newspapers and emails to local psychotherapists. Additionally, some patients were recruited via the psychotherapeutic outpatient department at the University of Münster, which offers a weekly consultation hour for patients who, if suitable, are proposed to participate in a clinical study in order to bypass the waiting time for a regular CBT treatment.

All data were collected at the psychotherapeutic outpatient department of the Christoph-Dornier-Foundation in Münster. Participants received € 30 each for the completion of both pre- and post-assessment as well as € 40 for completing follow-up (FU) assessment. Moreover, participants in the WL were paid additional € 20 for their participation in a second pre-assessment (Pre2). Beyond that, participants received an additional amount of € 80 to € 100 for filling in questionnaires of a smartphone-based ecological momentary assessment (EMA) study that was run prior to the first treatment session and directly after the last treatment session. Results of the EMA study will be reported elsewhere.

### Assessment

A two-step assessment was conducted to check inclusion/exclusion criteria. The first step involved a phone screening that was conducted by a graduate student research assistant. Second, participants meeting the criteria of the phone screening were invited to an assessment session (Pre1) which was conducted by one of six independent study evaluators. All evaluators were Master level psychologists currently participating in an advanced training to become a cognitive behavioral psychotherapist. They received special training in diagnosing OCD by the investigators. The evaluators were blind with regard to the treatment condition of the patient (DM vs. CR). Contrary to the protocol registered at clinicaltrials.gov, however, the evaluators could not be blinded in terms of whether the patient was in the WL or the NWL condition due to aspects concerning the organization of the study process. Participants were blinded in a way that they were not told about the contents of the other treatment condition until FU assessment in order to avoid any unintended mixing of treatment strategies.

**Fig 1:**
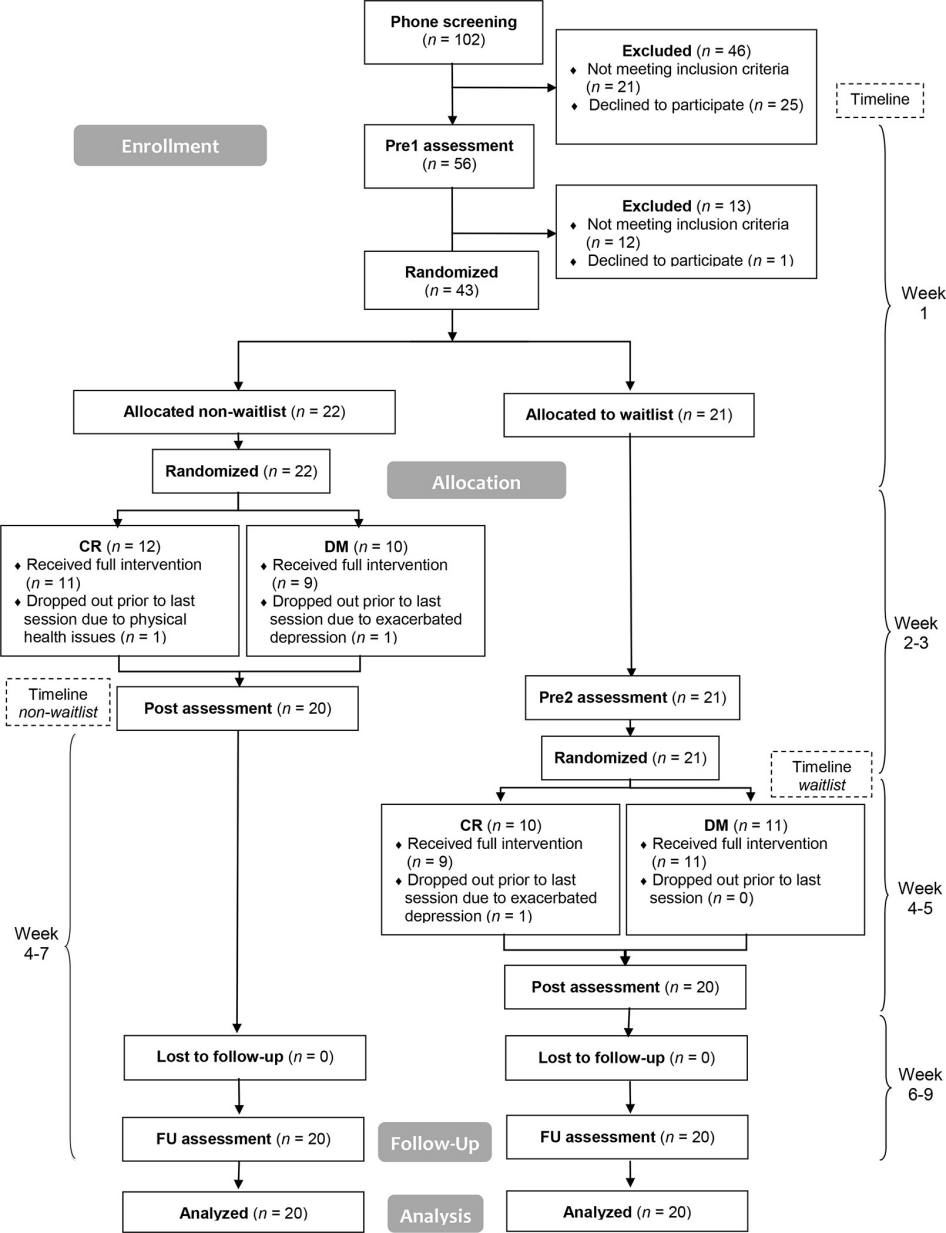
CONSORT flowchart describing the study process and participant flow. The reasons for exclusion after Pre1 assessment were as follows: OCD at subclinical level (*n* = 5), other than OCD being the primary diagnosis (*n* = 2), therapy focusing on OCD within the past 12 months (*n* = 2), history of psychosis (*n* = 1), recent change of medication (*n* = 1), acute Borderline Personality Disorder (*n* = 1), declined because experienced study protocol as too stressful (*n* = 1). *Abbreviations*: CR = cognitive restructuring, DM = detached mindfulness.

In sum, all participants underwent three assessments, i. e. Pre1, Post, and FU. Apart from that, the participants who were assigned to the WL condition during the first randomization received an additional assessment referred to as Pre2. The time span between Pre1 and Post (in the NWL group), between Pre1 and Pre2 (in the WL group) and between Pre2 and Post (in the WL group), respectively, was two weeks. The time span between Post and FU assessment was 4 weeks. Fig 1 is a a CONSORT flow diagram giving an overview of the study process and the assessment points.

Pre1 assessment comprised about three hours and included, among others, the administration of the German versions of the Structured Clinical Interview for DSM-IV Axis I Disorders (SCID-I) [30] as well as the German version of Y-BOCS [31]. The level of premorbid intelligence was assessed by the Multiple-choice vocabulary intelligence test (MWT) [32]. Information about demographic variables as well as previous pharmacological or psychotherapeutic treatments was collected. At the end of the Pre 1 assessment, participants filled in a number of questionnaires, most of which they additionally filled in prior to each treatment session to obtain process measures. Among these questionnaires was the German version of the Beck Depression Inventory-Revised (BDI-II) [33] measuring the intensity of depressive symptoms.

The primary outcome measure was the Y-BOCS total score (items 1–10). The primary outcome measure was assessed at three (NWL) or four (WL) time points. Baseline measures were collected during Pre1 assessment and, in the WL group, additionally during Pre2 assessment. Post assessment was conducted directly after the last treatment session. FU assessment was conducted 4 weeks after the end of the treatment. During the follow-up period no additional treatment was provided, but participants were told and encouraged to further practice the techniques they had learned in therapy.

### Inclusion and exclusion criteria

Inclusion criteria comprised a current primary diagnosis of OCD according to DSM-5 [1], based, however, on the German version of the SCID-I [30] complemented by additional questions to confirm the DSM-5 diagnosis. Further requirements comprised a minimum total Y-BOCS score of 16, a minimum age of 18 years, fluent German language skills and a minimum IQ of 80. Exclusion criteria included current suicidality or suicidal behavior within the past six months, a current or lifetime diagnosis of bipolar and psychotic disorder, a current addictive disorder and a current borderline personality disorder. Also, participants were excluded if they were currently undergoing cognitive-behavioral therapy focusing on OCD or if they had undergone such treatment in the past 12 months. For patients under medication, it was required that the dose was stable for at least eight weeks prior to Pre1 assessment. Patients not meeting these criteria were told to contact the investigators when their medication had been stable for at least eight weeks. Similarly, patients withdrawing from medication had to be at least eight weeks off their prior medication before entering the study.

### Randomization

Based on the total Y-BOCS score and the total score from the BDI-II [33] at baseline (Pre1) as well as age and sex, participants were randomized to either WL or NWL by minimization conducted with *MinimPy program 0*.*3* [34] using default settings. Either following Pre1 (NWL) or following Pre2 assessment (WL), participants were once more randomly allocated to the treatment group (CR/DM) using the same minimization procedure as described above. Allocation ratio was 1:1 for both randomizations. Whereas for NWL, this second randomization was based on the Pre1 scores of Y-BOCS and BDI-II, the Pre2 scores were used for the WL. Randomization was conducted by a graduate student research assistant.

### Treatment

Treatment in both conditions (DM/CR) comprised four sessions delivered within two weeks. Both therapists were Master level psychologists at an advanced stage of their clinical CBT licensing training. Supervision was provided by the fourth author (F. A.) and both therapists received special training in delivering DM and CR in the context of OCD prior to the start of the study. Participants were randomly assigned to one of the two therapists (C. R., C. J.).

Treatment consisted of two sessions per week, with a minimum of one day in-between the two weekly sessions. Each session lasted 100 min. The two treatment protocols were manualized by the first two authors drawing on the guidelines by Wilhelm and Steketee [35] for the CR group and those by Wells [6] for the DM group, respectively. The German study manuals can be provided upon request.

Based on the suggestions by the OCCWG, the CR procedure proposed by Wilhelm and Steketee [35] focuses on six cognitive domains, which are *overimportance of thoughts*, *control of thoughts*, *overestimation of danger*, *desire for certainty*, *responsibility*, and *perfectionism*. As *overimportance of thoughts* refers to thought action fusion and *control of thoughts* contains beliefs about the need to control thoughts—which both are parts of metacognitive models of OCD—these domains were explicitly excluded from the CR manual in order to avoid an overlap with metacognitive aspects and strategies. Similarly, the DM manual did not feature any references to a conceptual way of dealing with cognitions.

### Adherence and competence ratings

All treatment sessions were videotaped. For adherence and competence ratings, four complete treatments from each therapist (two DM and two CR, each) were randomly selected and then rated by an independent Master level psychologist using a list of items all employing a 5-point Likert scale, with 5 indexing the best score. The rater was at an advanced stage of his CBT licensing training and was not otherwise involved in the study. The rater had received training concerning both CR and DM prior to rating the video material.

### Cognitive restructuring

The first session of the CR condition consisted of psychoeducation about characteristics of OCD (e. g., the fact that obsessive thoughts are ubiquitous and therefore do not constitute the actual problem) and comprised the development of an individual cognitive model based on the model of Salkovskis [14, 15]. Focusing on the patient’s individual obsessive-compulsive symptoms, the model was designed to explain the disorder’s maintenance via the distorted appraisals of intrusions, which should therefore be questioned and altered. During the second session typical cognitive distortions occurring in OCD were explained (overestimation of danger, desire for certainty, responsibility and perfectionism) and strategies to question and modify these appraisals were provided and trained, drawing from Socratic questioning and logical and hedonistic strategies of disputation. Depending on the appraisal at hand, this would for instance include techniques such as multiplication of probabilities, distribution of responsibility, cost-benefit analysis, etc., whereas the whole process of questioning was recorded in writing. Prior to and after each questioning phase, the patient was asked to rate his/her level of conviction concerning the original appraisal. Additionally, s/he was encouraged to develop an alternative cognition and to practice this new cognition in everyday life. During the third session and the first part of the fourth session, these strategies were further trained. The last part of the fourth session consisted of summarizing the new knowledge and the techniques the patient had acquired during therapy. Homework during therapeutic sessions comprised monitoring and documenting obsessive thoughts and dysfunctional appraisals as well as engaging in the active questioning of the latter and practicing alternative cognitions in everyday life.

### Detached mindfulness

The first session of the DM condition comprised the same psychoeducation as in the CR treatment. Afterwards, therapist and patient developed an individual metacognitive model of the patient’s obsessive-compulsive symptoms (based on Wells [6]), explaining the maintenance of the disorder via the mechanism of assigning intrusive thoughts too much importance and meaning. That is, in contrast to the CR condition, patients were taught that it is about their general attitude towards their obsessions, not about any specific appraisals in response to their obsessions. During the second session, therapist and patient developed a list of the most prominent obsessions, rating each obsession’s frequency and its level of distress. This was followed by introducing the strategy of DM using different examples and metaphors and finally by training DM applying the suggestions by Wells [6]. Training DM usually involved the patients closing their eyes and following the therapist’s standardized suggestive instructions to visualize an obsession, to dissociate oneself from the obsession and to switch to a mode of passive observing instead of active interaction. Each training unit was designed to comprise five to ten minutes. The third session and the first part of the fourth session consisted of further DM training. The last part of the fourth session was identical to the CR condition. Homework between therapeutic sessions comprised monitoring and documenting obsessive thoughts (only between sessions 1 and 2) and exercising DM several times per day. Patients were instructed to practice DM on the obsessions previously included in their list and to then increasingly apply DM to all kinds of triggering thoughts they encountered in everyday life.

### Process measures

Prior to each treatment session, participants completed a number of questionnaires used as process measure that were presented on a tablet computer using a web-based online-survey software (www.unipark.de). Except for the results of the German version of the BDI-II [33], results of these process measures will be reported in a separate article.

### Homework ratings

During each session, homework compliance was rated by the therapist on a 7-point rating scale ranging from 1 (no homework implemented) to 7 (homework done exactly as the patient was told). The first ratings were obtained in the second session, referring to homework set in the first session.

### Data analysis

Data were analyzed using the *R* package *ez* [36] and *IBM SPSS Statistics (SPSS) 25*.*0*. Comparability of groups at baseline was analyzed by calculating independent *t*-tests for continuous variables and *χ*^2^-test for categorical variables. In order to evaluate the efficacy of treatments, a 2x2x2 mixed ANOVA was run with the between-subjects factors *waitlist* (WL/NWL) and *treatment condition* (CR/DM) and the within-subjects factor *time* coding whether measurements were taken at *T1* or *T2*: In both groups, *T1* referred to Pre1 data, however, *T2* referred to Pre2 data in the WL group and to Post data in the NWL group, thus separating effects of time and treatment resp. waitlist. Please note that (a) this mixed ANOVA does not use the Post assessment data from participants in the waitlist group, avoiding some implicit assumptions and complexities of linear mixed models [37], and that (b) the definition of time points therefore differs between WL and NWL groups. Please also note that (c) the F-test of the *waitlist*×*time* interaction tests the global treatment effect [38]. The use of the R package *ez* involved the computation of the *generalized eta squared statistic* ($\eta_{G}^{2}$) in order to display the amount of explained variance [39].

The calculation of effect sizes (Cohen’s *d*) was based on the complete data set including the Post data from WL participants, which was based on the a priori assumption of *time* not exhibiting a considerable main effect. Due to this assumption, Pre2 data were not used in the calculation of effect sizes. As the standard deviation of the outcome variable could be influenced by treatment resp. follow-up, Becker [40] recommends to use the value at the first time point. Three effect sizes (Pre1-Post, Post-FU, and Pre1-FU) were calculated for each treatment condition as follows, using pooled standard deviations (*SD*_*Pre*1_: 3.385; *SD*_*Post*_: 5.789) instead of separate standard deviations for CR and DM (*X* = mean Y-BOCS score, *SD* = standard deviation): *d*_*1*_ = $\frac{X_{Pre1}–X_{Post}}{{SD}_{Pre1}}$, *d*_*2*_ = $\frac{X_{Post}–X_{FU}}{{SD}_{Post}}$, *d*_*3*_ = $\frac{X_{Pre1}–X_{FU}}{{SD}_{Pre1}}$. Confidence intervals for Cohen’s *d* were calculated using the formula provided by Hedges and Olkin [41].

Moreover, in order to display the *amount* of improvement, clinically significant change was assessed as proposed by Jacobson and Truax [42]. It was defined by a combination of two criteria: (I) *reliable improvement*: $RC=\frac{X_{2}-X_{1}}{S_{diff}}$, with *x*_1_ representing a person’s Y-BOCS score at Pre1 assessment, *x*_2_ referring to a person’s Y-BOCS score at Post assessment, and *S*_*diff*_ denoting being the standard error of difference scores (based on the internal consistency of the German version of the Y-BOCS (*r* = .80) as reported by Jacobsen et al. [43]. Based on our calculations, a decrease of 5 points or more on the Y-BOCS indicated was used as an index of reliable improvement. (II) *recovery criterion*: *a* = *M*_1_−2*SD_1_, with *M*_1_ representing the mean Y-BOCS score of the sample at Pre1 assessment and SD_1_ referring to the corresponding standard deviation. A post assessment Y-BOCS score of *a* = 17.9 or less indicated recovery. A reliable change was presumed if participants displayed a post-assessment Y-BOCS score of 17.9 or less (recovery criterion) and a minimal Pre1-Post change of 5 -points on the Y-BOCS (reliable improvement criterion).

## Results

### Sample description

The recruitment process and participant flow is displayed in Fig 1. As you can see from the flowchart, three participants dropped out after randomization, two of which because of exacerbated depression and one due to physical health issues. Apart from this, there were no further adverse events or unintended side effects in any of the groups. Apart from the participants who dropped out, there was one missing data set of a CR participant concerning the process measures at FU assessment, which was due to a malfunctioning of the tablet used for data collection. There were no further missing data.

Regarding the three dropouts, an intention-to-treat (ITT) analysis was carried out under a missing at random (MAR) assumption employing multiple imputation by chained equations using predictive mean matching for the three missing continuous outcomes at T2 [44]. There were no substantial differences in any of the *p*-values of the repeated measures ANOVA in any of the 25 imputed datasets. Thus, the analyses described below are based on the completer sample (*n* = 40). By contrast, the report of the sample characteristics (Table 1) is based on the intent-to-treat (ITT) sample (*n* = 43).

**Table 1:** Demographic and clinical characteristics at Pre1 assessment (intention-to-treat sample).

Variable	NWL	WL	*p*	CR	DM	*p*
	(*n* = 22)	(*n* = 21)		(*n* = 22)	(*n* = 21)	
**Age**, mean (*SD*)	31.59	30.43	0.710	31.23	30.81	0.894
	(11.73)	(8.42)		(10.96)	(9.48)	
**Sex**, *n* (*%*)						
Male	9 (40.91)	9 (42.86)	1.000	12 (54.54)	6 (28.57)	0.124
Female	13 (59.09)	12 (57.14)		10 (45.45)	15 (71.43)	
**Family status**						
Single, *n* (*%*)	17 (77.27)	19 (90.48)	0.535	18 (81.82)	18 (85.74)	1.000
Married, *n* (*%*)	4 (18.18)	2 (9.52)		3 (13.63)	3 (14.29)	
Widowed, *n* (*%*)	1 (4.55)	0 (0.00)		1 (4.55)	0 (0.00)	
**Employment**						
Working full-time, *n* (*%*)	9 (40.91)	7 (33.33)	0.405	10 (45.45)	6 (28.57)	0.550
Working part-time, *n* (*%*)	6 (27.27)	10 (47.62)		7 (31.82)	9 (42.86)	
Not working, *n* (*%*)	7 (31.82)	4 (19.05)		5 (22.73)	6 (28.57)	
**Years of school**	13.11	13.07	0.954	12,57	13.64	0.139
**education**, mean (*SD*)	(1.09)	(3.14)		(1.03)	(3.06)	
**Clinical characteristics**						
Mean persistence of	10.32	12.19	0.506	10.36	12.14	0.527
OCD, years (*SD*)	(8.45)	(9.77)		(8.81)	(9.45)	
Mean age of onset, years	18.41	16.41	0.538	18.61	16.190	0.453
(*SD*)	(10.57)	(10.59)		(11.99)	(8.80)	
Number of comorbid	1.00	0.57	0.268	0.68	0.90	0.568
disorders, mean (*SD*)	(1.51)	(0.93)		(1.36)	(1.18)	
Number of previous	2.86	2.52	0.718	2.82	2.57	0.793
inpatient & outpatient	(3.09)	(3.04)		(3.10)	(3.04)	
treatments, mean (*SD*)						
Participants under	8	11	0.364	11	8	0.543
psychopharmacological	(36.36)	(52.38)		(50.00)	(38.10)	
medication, number (*%*)						
Participants experienced	1	3	0.345	3	1	0.607
in the intervention	(4.54)	(14.29)		(13.64)	(4.76)	
delivered, number (*%*)						

The number of participants experienced in the intervention delivered was determined by the therapists who asked participants during treatment whether they are familiar with the strategy, e. g. due to previous therapies. Fisher’s exact test was used for calculating comparisons for the variables *sex*, *family status*, *employment*, *Participants under psychopharmacological medication*, and *Participants experienced in the intervention delivered*, with the *p* value referring to a two-sided test. *t*-test for independent samples were computed for the remaining variables. All *p* values refer to comparisons between the groups listed in the two columns to the left, respectively.

Current comorbid disorders of the intention-to-treat sample were (percentage in brackets): Specific Phobia: 5 (11.63%), Alcohol Dependence Syndrome, in remission: 3 (6.98%), Major Depressive Disorder: 3 (6.98%), Social Anxiety Disorder: 3 (6.98%), Dysthymia: 2 (4.65%), Generalized Anxiety Disorder: 2 (4.65%), Post-traumatic Stress Disorder: 2 (4.65%), Body Dysmorphic Disorder: 2 (4.65%), Somatization Disorder: 2 (4.65%), Cannabinoid Dependence Syndrome, in remission: 1 (2.33%), Panic Disorder with Agoraphobia: 1 (2.33%), Panic Disorder without Agoraphobia: 1 (2.33%), Agoraphobia without Panic Disorder: 1 (2.33%), Undifferentiated Somatoform Disorder: 1 (2.33%), Persistent Somatoform Pain Disorder: 1 (2.33%), Trichotillomania: 1 (2.33%), Dermatillomania: 1 (2.33%), Overeating: 1 (2.33%), Attention Deficit Hyperactivity Disorder: 1 (2.33%). *Abbreviations*: NWL = non-waitlist, WL = waitlist, CR = cognitive restructuring DM = detached mindfulness.

Table 1 gives an overview of clinical and demographic characteristics along with between-group comparisons. We did not find any significant differences between WL and NWL and neither between CR and DM concerning any of the variables displayed (all *p*’s > .05). Y-BOCS and BDI-II data for the different assessment points across groups (completer sample) are presented in Table 2.

**Table 2:** Y-BOCS data, BDI-II data, and Cohen’s d (completer sample).

Variable	NWL	WL	*p*	CR	DM	*p*
	(*n* = 20)	(*n* = 20)		(*n* = 20)	(*n* = 20)	
**Y-BOCS** (items 1–10),						
mean (*SD*)						
Pre1	25.50	23.85	0.125	25.05	24.30	0.491
	(3.82)	(2.74)		(2.69)	(4.00)	
Pre2	-	23.60	-	-	-	-
		(2.39)				
Post	20.40	18.05	0.204	19.40	19.05	0.851
	(6.71)	(4.57)		(5.38)	(6.30)	
FU	-	-	-	16.35	17.05	0.797
				(9.11)	(7.92)	
**BDI-II**, mean (*SD*)						
Pre1				17.65	16.55	
				(9.29)	(10.66)	
Post				16.30	14.55	
				(10.39)	(12.71)	
FU (DM: *n* = 20,				12.00	13.10	
CR: *n* = 19)				(8.49)	(13.04)	
**Cohen’s *d***			**CR**	(*n* = 20)	**DM**	(*n* = 20)
(95% confidence intervals						
in square brackets)						
Pre1-Post			**1.67**	[0.95; 2.39]	**1.55**	[0.84; 2.26]
Post-FU			**0.53**	[-0.10; 1.16]	**0.35**	[-0.28; 0.98]
Pre1-FU			**2.57**	[1.73; 3.41]	**2.14**	[1.36; 2.92]

Cohen’s *d* is based on the Y-BOCS data (items 1–10). The calculation of *M* and *SD* for the BDI-II at FU in the CR condition was based on 19 instead of 20 participants due to one missing data set. The *p* values refer to *t*-tests for independent samples between the groups listed to the left. *Abbreviations*: NWL = non-waitlist, WL = waitlist, CR = cognitive restructuring DM = detached mindfulness.

### Homework ratings

The results of the homework ratings can be retrieved from Table 3.

**Table 3:** Homework ratings.

	CR (*n* = 20)	DM (*n* = 20)
Second session, mean (*SD)*	5.85 (1.39)	6.50 (0.69)
Third session, mean (*SD)*	5.45 (1.39)	5.45 (1.32)
Fourth session, mean (*SD)*	4.90 (1.68)	5.50 (1.43)

There are no homework ratings for the first session because the first homework was set at the end of the first session. *Abbreviations*: CR = cognitive restructuring DM = detached mindfulness.

### Efficacy of treatment

#### Mixed ANOVA

**Fig 2:**
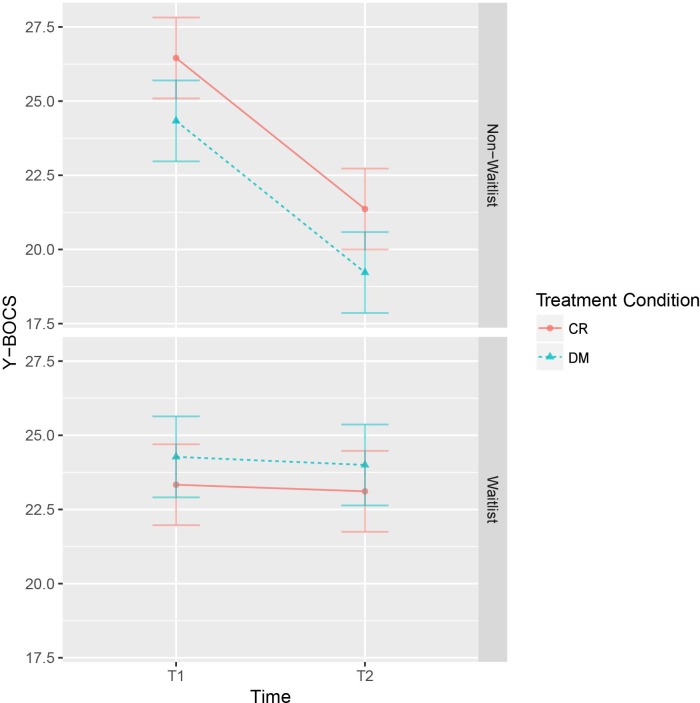
Line graph showing the results of the mixed ANOVA with 95% confidence intervals. The upper graph visualizes the results from the non-waitlist group of completers (total *n* = 20; CR: *n =* 11; DM: *n =* 9), whereas the lower graph displays the results from the waitlist group of completers (total *n* = 20; CR: *n =* 9; DM: *n =* 11). In both graphs, T1 refers to the Pre1 assessment. In the upper graph, T2 refers to the Post assessment, whereas in the lower graph, T2 refers to the Pre2 assessment, thus separating the effects of time and treatment. *Abbreviations*: CR = cognitive restructuring, DM = detached mindfulness.

The mixed 2x2x2 ANOVA used the Y-BOCS score as dependent variable since this was the a priori-defined outcome measure. The results of the mixed ANOVA are displayed in Table 4, whereas mean Y-BOCS-scores are displayed in Fig 2. The significant main effect for *time* (*p* < .001) indicates a global change in mean Y-BOCS scores across all combinations of *waitlist* and *treatment condition*s, while the non-significant main effects for *waitlist* and *treatment condition*s as well as the non-significant interaction of these two factors are interpreted as no difference at T1 (= Pre1), as expected by randomization and confirmed by Fig 2. The significant *waitlist* x *time* interaction (*p* = 0.001) indicates that mean Y-BOCS scores change from T1 to T2 when treatment is immediate (see upper panel of Fig 2), while the non-significant *treatment condition* x *time* and *waitlist* x *treatment condition* x *time* interactions correspond to parallel mean Y-BOCS score changes (see upper panel of Fig 2) for both treatment types, i. e., no change for waiting participants (see lower panel of Fig 2). In sum, the results are in line with our hypotheses, indicating no considerable effect of the time spent waiting in the waitlist condition and showing both treatment conditions to be similarly effective.

**Table 4:** Results of the mixed 2x2x2 ANOVA.

	*df*_*1*_	*df*_*2*_	*F*	*p*	*η*_G_^*2*^
*Time*	1	36	15.82	< 0.001	0.097
*Treatment condition*	1	36	0.26	0.611	0.005
*Waitlist*	1	36	0.50	0.486	0.010
*Waitlist*×*Time*	1	36	12.89	0.001	0.081
*Waitlist* ×*Treatment condition*	1	36	1.65	0.208	0.033
*Treatment condition* ×*Time*	1	36	0.001	0.979	< 0.001
*Waitlist*×*Treatment Condition* × ×*Time*	1	36	<0.001	0.991	< 0.001

The dependent variable for this ANOVA was the Y-BOCS score which served as the primary outcome measure. The degrees of freedom for the numerator of the *F* test are referred to as *df*_*1*,_ whereas the degrees of freedom for the denominator of the F test are referred to as *df*_*2*._ The *generalized eta squared statistic* ($\eta_{G}^{2}$) is used to display the amount of explained variance.

#### Effect sizes

As one can see from Table 2, we found large Pre to Post effect sizes concerning the Y-BOCS score for both treatment conditions, with a non-significant trends towards symptom severity even further decreasing between Post and FU assessment.

#### Clinical significant change

At Post assessment, a clinical significant change based on the criteria described above was evident in 8 (40%) patients from the DM group and in 8 (40%) patients from the CR group–based on the Y-BOCS score.

### Adherence and competence ratings

Across sessions and therapists, the mean adherence ratings were 4.78 (*SD* = 0.11) in the CR condition and 4.99 in the DM condition (*SD* = 0.02). Also across sessions and therapist, mean competence ratings were 4.70 (*SD* = 0.11) in the CR condition and 4.67 (*SD* = 0.05) in the DM condition.

## Discussion

Our results demonstrate that, in line with our expectations, both treatment conditions were superior to the waitlist condition concerning clinical improvement on the gold standard Y-BOCS. As the interaction effect for *time* and *waitlist* indicates, there was a significant symptom reduction in both treatment conditions relative to the WL condition. Regarding the effect sizes in both treatment conditions (DM: *d* = 1.55, CR: *d* = 1.67) and the fact that in both conditions, 40% of the patients exhibited a clinical significant change, our results are promising, especially in the light of the short treatment period. As such, our results confirm and extend the findings of Firouzabadi and Shareh [12], Ludvik and Boschen [13] and Wahl et al. [11] suggesting the efficacy of DM when intensely delivered as a stand-alone intervention in a clinical sample and under randomized controlled conditions. Also, the results concerning BDI-II point to a slight reduction of depressive symptoms across time (from Pre1 to Post to FU) in both the DM and the CR condition.

Moreover, it is worth mentioning that our findings concerning effect sizes and percentage of patients exhibiting a clinical significant change are not as different from those by Fisher and Wells [7], Rees and van Koesveld [8], Shareh et al. [9] and Simons et al. [10] as one might expect taking into account that treatment in those studies comprised between 10 and 20 weekly sessions and a large variety of metacognitive interventions beyond DM. In sum, our findings concerning the CM condition confirm the theoretical assumptions of the model put forward by Wells [6] and underline the crucial role of altering patients’ attitudes towards their inner events in reducing OCD symptoms.

Also with regard to the CR condition, our results contribute to the issue of whether behavioral experiments are necessary for making CR for OCD effective. Since we designed the CR condition analogously to the DM condition by limiting treatment to the purely Beckian elements, i. e., questioning of thoughts and beliefs and developing alternative cognitions, our findings offer the possibility of isolating those cognitive principles from the exposure-associated confounds of behavioral experiments, demonstrating that even within a very limited time frame, cognitive restructuring can lead to clinical significant change. Hence, the findings for the CR condition correspond to and extend those by Belloch et al. [29] showing that CR excluding behavioral experiment can also lead to considerable effect sizes in the treatment of OCD. Finally, our results further confirm the cognitive models of OCD developed by Salkovskis [14, 15] and Rachman [16].

However, this study only partly addressed the question which working mechanisms underlie the *detached mindfulness* technique and to what extent the two treatment conditions share common working mechanisms. The results concerning process measures of cognition and metacognition collected in this study will be reported elsewhere. Yet, our clinical experience while delivering treatment in the DM condition raised some hypotheses concerning underlying working mechanisms beyond those proposed by Wells [6]. Based on the observation that several patients reported heightened and decreasing feelings of distress and tension while applying DM to their obsessions, future research should focus on the question to what extent DM is actually similar to in sensu exposure, sharing, e. g., the working mechanisms of habituation, fear extinction, inhibitory learning and expectancy violation [45–47]. Moreover, future research should address the question whether DM can also be compared to a relaxation-based coping skill, since a large number of participants also reported experiencing the DM technique as relaxing and pleasant. Conceptually, this would place DM close to systematic desensitization [48]. What is yet to be investigated is the effectiveness of DM when applied in a non-randomized, uncontrolled clinical setting, since generalizability and external validity of our findings is clearly limited by factors such as the manualized treatment procedure and the short and intense treatment setting.

Besides, we would like to emphasize the conceptual overlap between DM and mindful-acceptance-based techniques, e.g., cognitive defusion, which form an important part of Acceptance and Commitment Therapy (ACT) [49]. To date, however, there are no studies on the efficacy of specific elements of ACT, whereas the efficacy of complex ACT treatment protocols has been demonstrated for OCD, e. g., [50]. Indeed, our results suggest that other techniques aiming at increasing a person’s distance to his or her thoughts, such as cognitive defusion, might also be effective as stand-alone techniques. Further research should therefore examine the relevance of these single treatment components within mindful-acceptance based therapies such as ACT to further clarify its relevance in the treatment of OCD.

All in all, this study exhibits a number of strengths. First, this trial was, to our knowledge, the first examining the efficacy of detached mindfulness as a stand-alone intervention, while comparing it to a purely cognitive treatment condition excluding any confounding elements such as behavioral experiments. Second, our sample can be regarded as representative of the clinical population with regard to e. g., symptom severity, disorder persistence, comorbidity, age and percentage of males/females, which are factors enhancing external validity. Third, adherence to the detailed protocol was ensured and shown to be very high in both conditions, just as competence ratings yielded very high scores, indicating a high quality of treatment. Fourth, our findings are useful for clinicians in a way that two interventions other than exposure and response prevention were shown to actually reduce OCD symptoms–including compulsions as measured with the Y-BOCS–without targeting the reduction of compulsions in the first place. We therefore would suggest to consider both DM and CR as strategies to pave the way for subsequent exposure treatment since they arguably mean less stress for the patient, thus having a lower risk of being refused.

Yet, the interpretation of our results is limited by various factors. First, the sample size was comparably small, so that statistical power was insufficient with regard to finding efficacy differences between the two treatment conditions, which, however, was not the goal of the study. It is a common phenomenon in psychotherapy outcome research that two active conditions are similarly effective, e. g. [51], however, the interpretation of our results is limited in a way that of course, they do not allow any conclusions concerning superiority or non-inferiority due to power issues. Second, the clinicians in charge of the diagnostic assessment were, due to organizational reasons, only blinded concerning the treatment condition, but not with regard to whether the participant was in the WL or in the NWL condition. Therefore, a certain bias towards the assumption of symptom improvement cannot be ruled out. Third, our study lacked a placebo condition, which is why the amount of change due to unspecific working mechanisms such as a good working alliance or gaining insight, e. g., [52], cannot be quantified. Similarly, both treatment conditions comprised psychoeducation and the development of an idiosyncratic maintenance model, which probably added to the treatment’s efficacy, as well. Fourth, it has to be taken into account that the treatment was delivered by only two therapists (i. e. the first two authors), which reduces the generalizability of our results. Last, our results are limited by the fact that, due to the FU time frame only comprising four weeks for organizational reasons, we are unable to make any statements about long-term efficacy.

## Conclusions

In sum, we were able to show that two conceptually very different treatment concepts relying on the intense training of a single technique within a limited time frame were effective at reducing OCD symptoms. As such, this study adds to the development of alternative effective treatment strategies for OCD. Taking all limitations into account, this study was the first to suggest that DM as a stand-alone intervention may be similarly effective as cognitive restructuring in treating OCD within a limited amount of time. However, future research is urgently needed to replicate our results, possibly in a larger sample, to address the underlying working mechanisms and to elucidate to what extent DM shares working mechanisms with other interventions such as ERP, relaxation and cognitive restructuring.

## Supporting information

S1 File CONSORT checklist. This checklist gives information on how this publication complies with the CONSORT guidelines.
(DOC)

S2 File Main data set. This Excel data set contains data on all variables relevant for the results reported in this publication.
(XLSX)

S3 File Adherence and competence ratings (DM). This Excel data set contains all adherence and competence ratings for the detached mindfulness condition.
(XLSX)

S4 File Adherence and competence ratings (CR). This Excel data set contains all adherence and competence ratings for the cognitive restructuring condition.
(XLSX)

S5 File Study protocol (Original). This document includes the original German study protocol that was approved of by the ethics committee.
(DOCX)

S6 File Study protocol (English translation). This document includes the English translation of the original German study protocol that was approved of by the ethics committee.
(DOCX)
