# Supplementary material for: A randomized waitlist-controlled trial comparing detached mindfulness and cognitive restructuring in obsessive-compulsive disorder
Source: PLoS One. 2019 Mar 20;14(3):e0213895. doi: 10.1371/journal.pone.0213895 (PMC6426247; doi:10.1371/journal.pone.0213895)
Supplement: S5 File — This document includes the original German study protocol that was approved of by the ethics committee. (DOCX) [file pone.0213895.s005.docx]

­­Antrag auf Stellungnahme der Ethikkommission

des FB 7: Psychologie und Sportwissenschaft

**1. Bezeichnung des Forschungsvorhabens**

Zum Umgang mit aufdringlichen Gedanken bei der Zwangsstörung – zwei Behandlungsstrategien im Vergleich

**2. Name und Kontaktdaten des Antragstellers (Dienstanschrift):**

| Prof. Dr. Ulrike Buhlmann |
| --- |
| AE Klinische Psychologie und Psychotherapie |
| Westfälische Wilhelms-Universität Münster |
| Fliednerstraße 21 |
| 48149 Münster |
| Tel.: +49(251) 83-34111 |
| Fax: +49(251) 83-31331 |
| Email: ulrike.buhlmann@uni-muenster.de |

**3. Angaben zu den Rahmenbedingungen des Vorhabens**

Eine Stellungnahme der Ethikkommission wird aufgrund der geplanten Publikation dieses Projektes und den Journalvorgaben bzgl. eines bestehenden Ethikvotums verlangt.

Geplant ist eine randomisierte Studie mit mehreren Messzeitpunkten. Bei der Untersuchung sollen psychometrische Werte von Personen mit Zwangsstörung erhoben werden. Die Teilnahme ist freiwillig und dauert 3 bis 5 Wochen (+ Follow-Up-Messung nach 1 Monat). Das Vorhaben wird in Kooperation mit der Christoph-Dornier-Stiftung in Münster durchgeführt und auch durch diese finanziert.

**4. Gegenstand und Verfahren des Vorhabens**

**Gegenstand.** Bei der Behandlung der Zwangsstörung wird die „störungsspezifische Kognitive Verhaltenstherapie (KVT) einschließlich Exposition und Reaktionsmanagement“ als Methode der Wahl empfohlen (Kordon, Lotz-Rambaldi, Muche-Borowski & Hohagen, 2013, S.37). Da jedoch ca. 30% der Patienten als Non-Responder gelten (Schruers, Koning, Luermans, Haack & Griez, 2005), scheint eine Weiterentwicklung der Therapieansätze lohnenswert, um den Therapieerfolg zu verbessern.

Neben der kognitiven Therapie (KT), auf deren Wirksamkeit bei der Behandlung von Zwangsstörungen bereits einige Studien hinweisen (Rosa-Alcázar, Sánchez-Meca, Gómez-Conesa & Marin-Martínez, 2008; Wilhelm et al., 2009; Belloch, Cabedo, Carrio & Larrsson, 2010; Olatunji et al., 2013), stellt die metakognitive Therapie (MKT) nach Wells (2011) einen weiteren Therapieansatz dar. Dabei steht die Veränderung der Beziehung der Patienten zu ihren Gedanken im Vordergrund. Diverse Studien scheinen auf die Wirksamkeit dieser Therapieform bei der Behandlung von Zwangsstörungen hinzuweisen (Bsp.: Shareh, Gharraee, Atef-Vahid & Eftekhar, 2010), wobei die angewandten Behandlungsprogramme aus einer Reihe verschiedener Einzeltechniken bestehen, sodass kein Rückschluss auf die Wirksamkeit spezifischer Einzelinterventionen gezogen werden kann. Die Erforschung der wirksamen Elemente etablierter Therapieprogramme erscheint im Hinblick auf die Steigerung von deren Effektivität jedoch sehr relevant (vgl. Johansson & Høglend, 2007). Im Rahmen der metakognitiven Therapie wird der Technik der *Detached Mindfulness* eine besondere Bedeutung zugesprochen (Wells, 2011), anhand derer die Patienten lernen sollen, vom so genannten *Objektmodus* in den *metakognitiven Verarbeitungsmodus* zu wechseln, wodurch die Beziehung der Pat. zu ihren Gedanken verändert werden soll. Bislang gibt es nur zwei Studien, die auf die Wirkung von Detached Mindfulness als Einzelintervention bei der Reduktion von Zwangssymptomen hinweisen (Bsp.: Ludvik & Boschen, 2015). Diese lassen aufgrund des Studiendesigns (Einzelfallstudie bzw. nicht klinische Population) jedoch nur bedingt Schlüsse über die Wirksamkeit zu.

Ferner ist noch weitgehend unklar, inwieweit sich die Wirkmechanismen zwischen kognitiven und metakognitiven Interventionen unterscheiden und ob für die Entstehung und Aufrechterhaltung einer Zwangsstörung eher kognitive oder metakognitive Überzeugungen relevant sind. Zwei Studien (Bsp.: Myers & Wells, 2005) legen nahe, dass Interventionen, die metakognitive Überzeugungen verändern, möglicherweise schneller zu Behandlungserfolgen führen als kognitive Interventionen.

Zur Sammlung ökologisch valider Daten (vgl. Ebner-Priemer & Trull, 2009) zum Verlauf einer Symptomatik bietet sich zudem die Methodik des Ecological Momentary Assessment (EMA) an, die jedoch unseres Wissens nach im Rahmen der Erforschung der Zwangsstörung erst zweimal eingesetzt wurde (Bsp.: Purdon, Rowa & Anthony, 2007). EMA-Daten zu kognitiven und metakognitiven Veränderungen während einer entsprechenden Intervention liegen zudem bislang nicht vor.

Die Studie zielt somit auf die Beantwortung der folgenden Forschungsfragen ab:

1. Führt die regelmäßige Anwendung der Detached Mindfulness zu einer sign. Reduktion der Symptome der Zwangsstörung (im Vgl. zu einer Wartelisten-Kontrollgruppe)?
2. Führt die regelmäßige Anwendung kognitiver Disputationstechniken bzgl. der Interpretation der Zwangsgedanken zu einer sign. Reduktion der Symptome der Zwangsstörung (im Vgl. zu einer Wartelisten-Kontrollgruppe)?
3. Wie gut gelingt es den Pat., die jeweilige Technik eigenständig effektiv einzusetzen?
4. Unterscheidet sich die Detached Mindfulness sign. von der Methode der kognitiven Umstrukturierung hinsichtlich der Reduktion von Häufigkeit und Intensität der Symptome der Zwangsstörung?
5. Lässt sich bereits durch die Psychoedukation eine sign. Reduktion der Symptomatik der Zwangsstörung erzielen?
6. Unterscheiden sich die unterschiedlichen Formen der Psychoedukation (MKT vs. KT) in ihrer Wirkung?
7. Wie verhalten sich die Symptome der Zwangsstörung (Häufigkeit von und Belastung durch Zwangsgedanken; Gefühle, die mit den Zwangsgedanken verbunden sind; Stärke des Dranges, auf Zwangsgedanken reagieren zu wollen; Verhalten in Reaktion auf den Zwangsgedanken) im Alltag der Patienten?
8. Wie verändert sich der Umgang mit intrusiven Gedanken im Rahmen der Interventionen (Häufigkeit der Anwendung der Strategie; Schwierigkeit der Anwendung; Entlastung durch die neue Strategie), sowohl hinsichtlich der Fragebogendaten als auch bzgl. der EMA-Daten?

**Methoden.** Die Datenerhebung wird einerseits über das Ausfüllen von Fragebögen stattfinden. Dieses erfolgt jeweils vor einer Sitzung mittels computergestützter Datenerhebung an einem Tablet (via Unipark). Die geplanten diagnostischen Erhebungen zu Beginn (Sitzung I & 1b) sowie am Ende der Studie (Sitzungen VI & VII) umfassen zudem ein klinisches Interview zur Diagnostik psychischer Störungen sowie ein Interview zur Erhebung des Schweregrades der Zwangsstörung. Außerdem wird im Rahmen der Prä-Diagnostik ein verbaler Intelligenztest durchgeführt. Die diagnostischen Erhebungen sowie die Einführung in die EMA-Erhebung werden von einem unabhängigen klinischen Diagnostiker durchgeführt. Hierbei wird es sich um PsychologInnen mit Master-Abschluss handeln, die sich in der Ausbildung zur psychologischen Psychotherapeutin/zum psychologischen Psychotherapeuten befinden. Folgende Fragebögen werden verwendet:

| **Messinstrument** | **Kurzbeschreibung** | **Sitzung Ia** | **Sitzung Ib** | **Sitzung II** | **Sitzung III** | **Sitzung IV** | **Sitzung V** | **Sitzung VI** | **Sitzung VII** |
| --- | --- | --- | --- | --- | --- | --- | --- | --- | --- |
| **Probandenanamnesebogen** | Geburtsdatum, Geschlecht, Staatsangehörigkeit, Familienstand, Kinder, höchster allgemeinbildender Schulabschluss, Alter bei Erwerb des höchsten allgemeinbildenden Schulabschlusses, Dauer der schulischen Ausbildung, höchster beruflicher Bildungsabschluss, Alter bei Erwerb des höchsten beruflichen Bildungsabschlusses, Dauer der nachschulischen Ausbildung, Erwerbssituation, Alter bei Beginn der Symptomatik, Alter bei Beginn der Belastung durch die Symptomatik, Suizidalität (aktuell und in der Vergangenheit), aktuelle psychiatrische Behandlung, aktuelle psychiatrische Medikation, psychotherapeutische Behandlung in der Vergangenheit, Anzahl der psychotherapeutischen Behandlungen in der Vergangenheit, Formen der psychotherapeutischen Behandlung(en), Datum der letzten Sitzung der Psychotherapie | √ | ---- | ---- | ---- | ---- | ---- | ---- | ---- |
| **Strukturiertes Klinisches Interview nach DSM-IV, Achse I** (SKID-I; Wittchen et al., 1997) | Strukturiertes Interview zur Diagnostik psychischer Störungen nach DSM-IV | √ | (nur Modul *Zwangs-störung*) | ---- | ---- | ---- | ---- | (nur Modul *Zwangs-störung*) | (nur Modul *Zwangs-störung*) |
| **Yale-Brown Obsessive Compulsive Scale, deutsche Version** (Y-BOCS; Hand & Büttner-Westphal 1991) | Halbstrukturiertes Interview zur Beurteilung des Schweregrads von Zwangsgedanken und –handlungen, wobei in dem Interview zeitlich auf die letzte Woche Bezug genommen wird. | √ | √ | ---- | ---- | ---- | ---- | √ | √ |
| **Yale Brown Obsessive-Compulsive Scale, Selbstabfrage** (Baer, 1993) | Selbstbeurteilungsfragebogen zur Erfassung des Schweregrads von Zwangsgedanken und –handlungen | √ | √ | ---- | √ | √ | √ | √ | √ |

**√** = wird in der Sitzung durchgeführt / **----**  = wird in der Sitzung nicht durchgeführt

| **Beck-Depressions-Inventar Revision** (BDI-II; Hautzinger et al. 2006) | Selbstbeurteilungsfragebogen zur Erfassung des Schweregrads von depressiven Symptomen in den letzten zwei Wochen anhand von insgesamt 21 Items | √ | √ | ---- | √ | √ | √ | √ | √ |
| --- | --- | --- | --- | --- | --- | --- | --- | --- | --- |
| **Mehrfachwahl-Wortschatz-Intelligenztest** (MWT; Lehrl, 2005) | Verbaler Leistungstest zur Erfassung des allgemeinen Intelligenzniveaus | √ | ---- | ---- | ---- | ---- | ---- | ---- | ---- |
| **Obsessive-Compulsive Inventory-Revised, deutsche Version** (OCI-R; Gönner, Leonhart & Ecker, 2007) | Selbstbeurteilungsfragebogen zur Erfassung des Schweregrads, der Hauptsymptome und Subtypen der Zwangsstörung auf 6 Subskalen (*Waschen, Kontrollieren, Ordnen, Zwangsgedanken, Horten, Neutralisieren mit Zahlen*) anhand von insgesamt 18 Items (Beispielitem: „Ich bewahre so viele Gegenstände auf, dass sie mich behindern.“) | √ | √ | ---- | √ | √ | √ | √ | √ |
| **Thought-Fusion-Instrument,** deutsche Übersetzung (TFI; Wells, Gwilliam & Cartwright-Hatton, 2002): | Selbstbeurteilungsfragebogen zur Erfassung der Ausprägung von Überzeugungen über die Bedeutung und die Macht von Gedanken; Erfassung der 3 Subskalen (*Thought Event Fusion, Thought Action Fusion, Thought Object Fusion*) anhand von insgesamt 14 Items (Beispielitem: „Wenn ich an ein unerfreuliches Ereignis denke, wird es dadurch mit einer höheren Wahrscheinlichkeit passieren.“) | √ | √ | ---- | √ | √ | √ | √ | √ |
| **Beliefs About Rituals Inventory**, deutsche Übersetzung (BARI; Wells & McNicol, 2004) | Selbstbeurteilungsfragebogen zur Erfassung der Ausprägung von positiven Überzeugungen über Rituale: deutsche Übersetzung des mit insgesamt 12 Items (Beispielitem: „Ich muss meine Rituale durchführen, sonst werde ich nie meinen inneren Frieden haben.“) | √ | √ | ---- | √ | √ | √ | √ | √ |
| **Stop Signals Questionnaire**, deutsche Übersetzung (SSQ; Myers, Fisher & Wells, 2009) | Selbstbeurteilungsfragebogen zur Erfassung der Ausprägung von Überzeugungen über Kriterien, die darüber entscheiden, wann man Rituale beenden kann: deutsche Übersetzung mit insgesamt 12 Items (Beispielitem: „Ein wichtiges Signal dafür, wann ich mit meinen Ritualen aufhören kann, ist, wenn ich meine Rituale in der richtigen Reihenfolge ausgeführt habe.“) | √ | √ | ---- | √ | √ | √ | √ | √ |
| **Obsessive-Beliefs Questionnaire,** deutsche Version (OBQ-D; Ertle et al., 2008) | Selbstbeurteilungsfragebogen zur Erfassung von Einstellungen und Überzeugungen gegenüber Intrusionen; deutsche Übersetzung; Erfassung der 3 Subskalen (Bedeutsamkeit von Gedanken/ Notwendigkeit, Gedanken zu kontrollieren, Perfektionismus/ Unsicherheitsintoleranz, Gefahrenüberschätzung/ Überhöhte subjektive Verantwortlichkeit) anhand von insgesamt 24 Items (Beispielitem: „ Die Dinge sollten, meinen eigenen Standards entsprechend, perfekt sein.“) | √ | √ | ---- | √ | √ | √ | √ | √ |
| **Personality Inventory for DSM–5**, deutsche Version (PID-5; Krueger, Derringer, Markon, Watson & Skodol, 2012) | Selbstbeurteilungsfragebogen zur Erfassung pathologischer Persönlichkeitszüge; deutsche Übersetzung mit insgesamt 100 Items (Beispielitem: „ Nichts scheint mich wirklich zu interessieren.“) | √ | ---- | ---- | ---- | ---- | ---- | ---- | ---- |
| **Hausaufgabenrating** | Selbstbeurteilungsfragebogen zur Erfassung der subjektiven Einschätzung bzgl. Durchführbarkeit und Effektivität der im Rahmen des Trainings gelernten Strategie (Beispielitem: „Wie schwer ist es Ihnen gefallen, die neu erlernte Technik anzuwenden?“) | ---- | ---- | ---- | ---- | ---- | √ | √ | √ |
| **Patient Questionnaire on Therapy Expectation and Evaluation,** deutsche Version (PATHEV; Schulte, 2005) | Selbstbeurteilungsfragebogen zur Messung der Therapieerwartung und Therapieevaluation anhand von 11 Items (Beispielitem: „ Ich glaube, dass meine Probleme jetzt endlich gelöst werden konnten.“) | ---- | ---- | ---- | ---- | ---- | ---- | √ | √ |

An zwei Zeitpunkten findet zudem jeweils eine EMA-Erhebung statt (nach Sitzung II und VI). Dabei erhalten alle Teilnehmer für den Zeitraum von jeweils 4 Tagen (Freitag bis Montag) ein Smartphone mit Touchscreen, das so programmiert ist, dass die Teilnehmer es lediglich für die Datenerhebung im Rahmen der Studie nutzen können. Zu 10 Zeitpunkten am Tag (diese sind zufällig ausgewählt, mit einem Mindestabstand von 30 Minuten zwischen den einzelnen Messzeitpunkten) gibt dieses Smartphone einen Signalton ab, welcher für den Teilnehmer eine kurze Befragung von ca. 2 Min. Dauer ankündigt.

Innerhalb von 15 Minuten nach dem Signalton kann der Teilnehmer die Befragung auf dem Smartphone durchführen. Reagiert er innerhalb dieser Zeitspanne, während der er insgesamt fünfmal durch einen Signalton zur Dateneingabe aufgefordert wird, nicht, entfällt dieser Befragungszeitpunkt. Diese Frist von 15 Minuten beruht auf den deutlichen Hinweisen von Delespaul (1995), dass 15 Minuten nach dem Signal die Reliabilität der Antworten sinkt, sodass diese Antworten nicht mehr in die Analysen einbezogen werden sollten. Der Teilnehmer hat zudem bei jedem Signalton die Möglichkeit, durch Drücken des entsprechenden Buttons auf dem Bildschirm den Messzeitpunkt zu verschieben oder den Messzeitpunkt ganz ausfallen zu lassen. Auch zwischen den Signaltönen kann der Teilnehmer durch Drücken eines entsprechenden Buttons („Bitte nicht stören“) das Smartphone für eine Stunde stumm schalten, sodass in dieser Zeit kein Signalton ertönt.

Auf 7-stufigen Likert-Skalen werden bei der EMA-Erhebung folgende Aspekte erfasst:

Nach Sitzung II („Prä-EMA“):

- Stressniveau seit dem letzten Signal
- Entspannungsniveau seit dem letzten Signal
- Häufigkeit der Zwangsgedanken seit dem letzten Signal
- Belastung durch die Zwangsgedanken seit dem letzten Signal
- Mit den Zwangsgedanken verbundene Gefühle
- Häufigkeit von bestimmten Reaktionsweisen auf die Zwangsgedanken seit dem letzten Signal

Nach Sitzung VI („Post-EMA“): Dieselben Items wie in der Prä-EMA-Erhebung, außerdem:

- Häufigkeit der Anwendung der neu erlernten Technik im Umgang mit Zwangsgedanken seit dem letzten Signal
- Subjektive Schwierigkeiten bei der Anwendung der neu erlernten Technik seit dem letzten Signal
- Subjektive Entlastung durch die Anwendung der neu erlernten Technik seit dem letzten Signal

Außerdem werden in beiden EMA-Erhebungen die Teilnehmer jeweils am Ende gebeten, zusätzlich anzugeben, welche Personen (bekannte/fremde) sich seit dem letzten Signal in ihrer Nähe befunden haben.

Im Anhang sind alle Items im Wortlaut dargestellt. Eine Pilotstudie mit 3 psychisch gesunden Probanden und insg. 83 beantworteten Signalen ergab eine mittlere Bearbeitungsdauer von 1:28 Minuten, sodass die Einschätzung der Bearbeitungszeit pro Signal auf etwa 2 Minuten realistisch erscheint.

**Experimentelle Aufgaben.** Finden nicht statt.

**Durchführung.** Die Probanden werden über die Ambulanz für Zwangsstörungen der Universität Münster (Leitung: Prof. Ulrike Buhlmann) sowie über die Christoph-Dornier-Stiftung Münster (Institutsleitung: Dr. Fabian Andor) rekrutiert. Weitere Patienten sollen über Aushänge und Annoncen rekrutiert werden.

Anschließend erfolgt ein erster telefonischer Kontakt, bei dem ein kurzes Telefonscreening durchgeführt wird (s. Anhang). Sofern die Teilnehmer im Telefonscreening keine Ausschlusskriterien erfüllen, werden sie zu einem persönlichen Termin eingeladen.

Die anschließenden Sitzungen finden an sieben Terminen (bzw. acht Terminen in der Wartelistengruppe) statt. Sitzung VI umfasst sowohl den Termin für die letzte Therapiesitzung als auch für die Post-Messung, die direkt im Anschluss an die letzte Therapiesitzung stattfindet, jedoch nicht vom Therapeuten, sondern von demselben unabhängigen Diagnostiker durchgeführt wird, der bei dem jeweiligen Teilnehmer die Prä-Messung in Sitzung Ia (und ggf. Ib) durchgeführt hat. Die Therapiesitzungen (Sitzungen III bis VI) dauern jeweils 100 Minuten, für Sitzung VI sollten die Teilnehmer wegen der anschließenden Post-Messung ca. 180 Minuten einplanen. Auch Sitzung Ia dauert ca. 180 Minuten, für Sitzung Ib und Sitzung VII sind hingegen 90 Minuten eingeplant, was eine großzügige Schätzung darstellt. Für die Einführung in die Handhabung des EMA-Smartphones sind aufgrund der ausführlichen Erklärungen zu den Items, die vom Diagnostiker an die individuellen Problembereiche des Teilnehmers angepasst werden, 45 Minuten veranschlagt. Zu Beginn einer jeden Sitzung werden die Probanden gebeten, die o.g. Diagnostik auszufüllen. Um Eingabefehler zu vermeiden, erfolgt das Ausfüllen der Fragebögen computergestützt an einem Tablet (via Unipark). Der Inhalt der Sitzungen ist in der folgenden Übersicht skizziert:

**Schematische Darstellung des Ablaufs:**

**SITZUNG I (Diagnostische Sitzung; Durchgeführt von Diagnostiker):**

- Ausgabe der Teilnehmerinformation und Ausfüllen der Einwilligungserklärungen
- Erhebung der demographischen Daten anhand des Probandenanamnesebogens
- Durchführung SKID-I , Y-BOCS sowie MWT
- Ausfüllen der Fragebögen
- Parallel: weitere Überprüfung der Ein- und Ausschlusskriterien

**Randomisierte Gruppenzuweisung und Mitteilung der Zuordnung per Telefon:** Teilnehmer der Wartelistenkontrollgruppe (WL) pausieren 2 Wochen; anschließend erfolgt erneute Prä-Diagnostik (*Sitzung Ib*)

**SITZUNG Ib (Nur WL; durchgeführt von Diagnostiker):**

- Durchführung der Sektion „Zwangsstörung“ des SKID-I sowie der Y-BOCS
- Ausfüllen der Fragebögen

**SITZUNG II (EMA-Einführung; durchgeführt von Diagnostiker):**

- Einführung in die Handhabung des EMA-Smartphones

**SITZUNG III (Durchgeführt von Therapeut):**

- Ausfüllen der Fragebögen
- Psychoedukation zur Zwangsstörung
- Erarbeitung eines je nach Bedingung kognitiven oder metakognitiven

Aufrechterhaltungsmodells der Zwangsstörung

- Je nach Bedingung: Ableitung der Strategien der kognitiven Umstrukturierung

bzw. der Detached Mindfulness

**SITZUNG IV (Durchgeführt von Therapeut):**

- Ausfüllen der Fragebögen
- Einführung der kognitiven Umstrukturierung/der Detached Mindfulness
- Einüben der kognitiven Umstrukturierung/der Detached Mindfulness

**SITZUNG V (Durchgeführt von Therapeut):**

- Ausfüllen der Fragebögen
- Einüben der kognitiven Umstrukturierung/der Detached Mindfulness

**SITZUNG VI (Durchgeführt von Therapeut & Diagnostiker):**

- Ausfüllen der Fragebögen
- Resümee bzgl. der Wirksamkeit der erlernten Strategie
- Entwicklung einer wirksamen Rückfallprophylaxe
- Durchführung Y-BOCS sowie Sektion „Zwangsstörung“ des SKID-I Interviews
- Ausfüllen des PATHEV
- Erläuterungen zu den drei neuen EMA-Items und Ausgabe des Smartphones

**SITZUNG VII (Durchgeführt von Diagnostiker)**

- Durchführung Y-BOCS sowie Sektion „Zwangsstörung“ des SKID-I Interviews
- Ausfüllen der Fragebögen
- Teilnehmer erhalten ihre Vergütung

**Körperliche Beanspruchung.** Keine körperliche Beanspruchung.

**Mentale Beanspruchung.** Die mentale Beanspruchung der Teilnehmer ist mittelgradig und ergibt sich vor allem durch die für die Teilnahme an der Diagnostik und an den für die therapeutischen Sitzungen benötigte Konzentrationsfähigkeit.

Die Teilnehmer beantworten zudem Fragen zu Bereichen, die für sie belastend sein können. Während der Diagnostik liegt daher ein Fokus darauf, die Belastung aufzufangen und die Eignung für die Studienteilnahme genau abzuwägen. Aus diesem Grund finden alle Kontakte zu den Teilnehmern mit psychotherapeutisch geschulten Versuchsleitern statt, die sich in der fortgeschrittenen Ausbildung zum Psychotherapeuten befinden und die während der Durchführung für telefonischen Kontakt bei Belastung zur Verfügung stehen. Sollten diese nicht erreichbar sein, können die Teilnehmer eine Nachricht auf dem Anrufbeantworter hinterlassen, ein Rückruf erfolgt innerhalb der Arbeitszeiten schnellstmöglich. Außerdem können die Probanden unter einer E-Mail-Adresse, die in den Teilnehmerinformationen angegeben ist, Kontakt zu den Versuchsleitern aufnehmen. Diese E-Mail-Adresse wird werktags innerhalb von 48 Stunden abgerufen, und Fragen werden zeitnah beantwortet. Für den Fall auftretender akuter Selbst- und Fremdgefährdung steht intern ein dezidierter Notfallplan der Psychotherapie-Ambulanz der Universität Münster bereit. Alle Mitarbeiter sind mit dem beschriebenen Prozedere vertraut und initiieren im Ernstfall die notwendigen Schritte bzw. wenden sich unmittelbar an die Versuchsleitung.

Durch die Smartphone-basierte Erhebung ist gemäß der aktuellen Reviews zum Ecological Momentary Assessment von Palmier-Claus et al. (2011) sowie Trull und Ebner-Priemer (2013) selbst bei schweren psychischen Störungen wie Schizophrenie, Depressionen und emotional-instabiler Persönlichkeitsstörung grundsätzlich nicht von einer zusätzlichen Beanspruchung auszugehen.

**Preisgabe persönlicher Informationen.** Wir erheben soziodemographische Variablen, Informationen zu psychischen Erkrankungen und Behandlungen und Fragebogenmaße zu Zwangssymptomen. Die Erhebung dieser persönlichen Informationen ist für die Studie unabdingbar. Die Informationen sind durch ein pseudonymisiertes Vorgehen den Teilnehmern nach der Erhebungsphase nicht mehr zuzuordnen.

**Täuschung und Aufklärung.** Die Teilnehmer werden vor Abgabe des Einverständnisses zur Teilnahme vollständig über den Zweck der Untersuchung sowie die Abläufe der Studie aufgeklärt, d.h. es kommt zu keiner Täuschung der Teilnehmer.

**5. Angaben zu Aufzeichnung, Aufbereitung, Speicherung und Löschung der Daten**

**Personenbezogene Daten.** Soziodemographische Daten werden von allen Teilnehmern erhoben. Die Namen der Teilnehmer werden ausschließlich zur Dokumentation des Einverständnisses zur Teilnahme an der Studie sowie im Rahmen der Kodierliste (s.u.) verwendet.

**Datenschutz.** Entsprechend § 3 Absatz 1 des Bundesdatenschutzgesetzes werden so wenig personenbezogene Daten wie nötig erhoben, verarbeitet und gespeichert. Die Diagnostik wird pseudonymisiert, d. h. in namentlich nicht gekennzeichneter Form unter einer Chiffre gespeichert. Es existiert eine schriftliche Kodierliste auf Papier, die den Namen mit der jeweiligen Chiffre verbindet. Die Kodierliste ist nur den Versuchsleitern zugänglich, die unter Schweigeplicht stehen. Jeder an der Studie beteiligte Studierende/Mitarbeiter unterschreibt zu diesem Zweck eine Schweigepflichterklärung.

Die computergestützte Datenerhebung erfolgt ohne Angabe von persönlichen Daten über Unipark, der Online-Befragungssoftware von Questback.

Über das Smartphone selbst kann nur mit dem Administratorkennwort, welches nur die zuständigen Mitarbeiter kennen, ein Zugriff auf die bisherigen gespeicherten Daten erfolgen. Die pseudonymen Daten auf dem Smartphone können ohne Kodierliste nicht auf die Person zurückgeführt werden. Sollte ein Smartphone eines Teilnehmers verloren gehen oder gestohlen werden, kann dieses zusätzlich über den Android Device Manager auf seine Werkseinstellungen zurückgesetzt werden, sodass alle Daten auf dem Smartphone gelöscht werden.

**Kodierliste.** Die Kodierliste wird in einem abschließbaren Schrank aufbewahrt und nach Ab-schluss der Datenverarbeitung, aber frühestens nach einem Jahr vernichtet. Nach Vernichtung der Kodierliste werden die anonymisierten Daten mindestens 10 Jahre gespeichert (ein Rückschluss auf die einzelnen Teilnehmer ist dann nicht mehr möglich). Die Teilnehmer werden gefragt, ob sie damit einverstanden sind, für spätere wissenschaftliche Untersuchungen noch einmal kontaktiert zu werden, und können sich in der Einverständniserklärung auch ohne Angabe von Gründen dagegen aussprechen.

**Löschung der Daten.** Solange die Kodierliste existiert, können die Probanden jederzeit die Löschung aller von Ihnen erhobenen Daten verlangen. Die Kodierliste wird nach Abschluss der Datenverarbeitung, aber frühestens nach einem Jahr vernichtet.

**6. Gewinnung der Personenstichprobe und Vergütung von Teilnehmern**

**Rekrutierung.** Die Rekrutierung der Stichprobe erfolgt über die Ambulanz für Zwangsstörungen der Universität Münster (Leitung: Prof. Ulrike Buhlmann) sowie über die Christoph-Dornier-Stiftung Münster (Institutsleitung: Dr. Fabian Andor). Weitere Patienten sollen über Aushänge und Annoncen rekrutiert werden.

**Personenstichprobe aus Datenbank?** Es wird keine Datenbank zur Rekrutierung verwendet.

**Merkmale der Personenstichprobe.** Es sollen ca. 60 Personen rekrutiert werden, die aktuell an einer klinischen Zwangsstörung leiden. In die geplante Studie werden Personen ab dem 18. Lebensjahr eingeschlossen.

**Einschluss- und Ausschlusskriterien.** Einschlusskriterium: Diagnose einer aktuellen klinischen Zwangsstörung als Primärdiagnose (basierend auf der aktuellen Symptombelastung/-schwere); bei Medikation: stabile Dosis seit mind. 8 Wochen

Ausschlusskriterien:

- Eine andere psychische Erkrankung als Primärdiagnose
- Alter < 18 Jahren
- Keine fließende Deutschkenntnisse
- Verbaler IQ < 80 (da hierbei die kognitiven Voraussetzungen für die Studienteilnahme nicht als gegeben angenommen werden können)
- Akute Suizidalität oder suizidales Verhalten in den letzten 6 Monaten
- Akute Psychose oder gesicherte Diagnose einer Erkrankung aus dem schizophrenen Formenkreis (F2) (aktuell oder in der Vergangenheit)
- Akute manische Episode
- Aktuelle Abhängigkeitserkrankung
- Akute Borderline-Persönlichkeitsstörung
- Aktuelle psychotherapeutische Behandlung bzw. psychotherapeutische Behandlung, bei der zwangsstörungsspezifische KVT-Interventionen durchgeführt wurden, im letzten Jahr
- Veränderung der Substanz oder der Dosis einer psychiatrischen Medikation während der letzten 8 Wochen bzw. während der Studie

**Internetbasierte Datengewinnung.** Die Teilnehmer füllen die Fragebögen über die Internetplattform Unipark aus (s.o.). Während dieser Zeit ist der Diagnostiker/der Therapeut für die Teilnehmer bei Fragen ansprechbar, sodass etwaige Unklarheiten und eventuell auftretende Schwierigkeiten bei der Touchscreen-basierten Dateneingabe bei damit nicht vertrauten Teilnehmern direkt aufgefangen werden können.

**Teilnahmevergütung**. Die Teilnahme an der Prä- und Post-Diagnostik wird mit jeweils 30€, die Teilnahme an der Follow-Up-Diagnostik wird zur Reduktion des Drop-out-Risikos mit jeweils 40€ vergütet. Die Teilnehmer, die der Wartelistenkontrollgruppe zugeordnet werden, erhalten bei der zweiten Prä-Diagnostik zudem weitere 20€ für ihre Teilnahme.

Die Vergütung wird den Teilnehmern nach der Follow-Up-Messung ausgezahlt. Bei Studienabbruch werden die Teilnehmer entsprechend ihrem bis dahin geleiteten Zeitaufwand im Rahmen der Diagnostik-Messungen vergütet. Die Teilnehmer quittieren den Erhalt der Vergütung.

Das Ausfüllen der EMA-Items wird pro Erhebungsphase zudem mit jeweils 40€ vergütet. Bei der Beantwortung von mindestens 80% der Smartphone-Signale erhalten die Teilnehmer zusätzlich eine Bonuszahlung in Höhe von 20€. Es wird angenommen, dass die Bonuszahlung für alle Teilnehmer unabhängig von demographischen oder psychopathologischen Merkmalen erreichbar ist. Um einen realistischen Eindruck über den Verlauf der interessierenden Variablen im Alltag zu erhalten, werden daher grundsätzlich keine individuellen Anpassungen diesbezüglich vorgenommen. Vor Beginn der Smartphone-basierten Erhebungsphase werden jedoch individuell relevante schwierige Situationen (z.B. Umgang mit dem Smartphone am Arbeitsplatz) exploriert und mögliche Umgangsstrategien abgewogen (z.B. vertraute Personen in Studienteilnahme einweihen, Auslassen einzelner Messzeitpunkte). Der Einfluss einer im Hinblick auf die Bonuszahlung möglichen Benachteiligung von in Vollzeit arbeitenden Teilnehmern ist dadurch deutlich reduziert, dass 50% der Erhebung am Wochenende, d.h. samstags und sonntags, stattfinden.

In den veröffentlichten EMA-Studien mit vergleichbaren Studiendesigns mit erwachsenen Teilnehmern ist die durchschnittliche Compliance stets sehr hoch (vgl. Review Trull & Ebner-Priemer, 2013), sodass davon ausgegangen wird, dass eine Compliancerate von 80% auch in der vorliegenden Studie z.B. trotz Berufstätigkeit (und Schichtarbeit) erreicht werden kann. Zudem ist ein derartiger Bonus in der Praxis üblich, um die Complianceraten zu erhöhen, z.B. Engel et al. (2013) 50$ Bonus bei 80% Compliance.

Beispiele für Complianceraten:

- Engel et al. (2013): durchschnittliche Compliance von 87% bei n=118 Probanden mit klinischer und subklinischer Anorexie. Gemäß durchgeführter Analysen war die Compliancerate nicht beeinflusst durch demographische Merkmale außer dem Alter.
- Goldschmidt et al. (2014): durchschnittliche Compliance von 86% und 75% innerhalb von 20 Minuten nach dem Signal bei n=133 Probanden mit Bulimie
- Kramer et al. (2014): durchschnittliche Compliance von 74% bei n=515 Frauen aus einer Zwillingsstichprobe (60% berufstätig)
- Mills et al. (2014) durchschnittliche Compliance von 84% in einer Stichprobe mit n=128 Frauen (39% vollzeitbeschäftigt, 36% teilzeitbeschäftigt)
- Santangelo et al. (2014): durchschnittliche Compliance von 94% bei n=43 Probanden mit Borderline-Störung, n=28 mit PTBS, n=20 mit Bulimie und 28 psychisch gesunden Kontrollen

**7. Freiwilligkeit der Teilnahme und Rücktritt**

**Freiwilligkeit.** Maßnahmen zur Sicherstellung der Freiwilligkeit sind gegeben. Beim ersten Termin erhalten die Teilnehmer ein Aufklärungsblatt zu den Hintergründen der Studie, in dem ausdrücklich auf die Freiwilligkeit der Teilnahme hingewiesen wird. Sie haben ausreichend Zeit sich die Entscheidung für oder gegen eine Teilnahme zu überlegen. Die Teilnehmer werden explizit darüber aufgeklärt, dass sie keine Vorteile durch die Studienteilnahme erwarten können sowie ihnen auch keine Nachteile bei Nichtteilnahme entstehen.

**Rücktritt.** Die Teilnehmer werden ausdrücklich darüber aufgeklärt, dass sie jederzeit ohne Nachteile von der Teilnahme an der Studie zurücktreten können. Sie werden darüber hinaus darüber aufgeklärt, dass die Erhebung der Fragebogendaten pseudonymisiert erfolgt, d. h. in namentlich nicht gekennzeichneter Form. Sie wissen, dass bis zum Abschluss der Datenerhebung eine Kodierliste existiert, die den Studienleitern zugänglich ist und nach Abschluss der Datenerhebung vernichtet wird. Die Teilnehmer wissen, dass sie einer Löschung ihrer Daten verlangen können, solange die Kodierliste existiert und dass dieses mindestens ein Jahr lang der Fall sein wird. Außerdem werden die Teilnehmer darüber aufgeklärt, dass sie auch bei Studienabbruch regulär auf die Warteliste der Christoph-Dornier-Stiftung oder der Psychotherapie-Ambulanz aufgenommen werden können, wenn sie weiterhin eine psychotherapeutische Behandlung wünschen.

**8. Umgang mit Zufallsbefunden**

**Aufklärung.** Körperliche Zufallsbefunde sind in der Studie nicht zu erwarten. Es werden psychologische Diagnosen im Rahmen der Diagnostik gestellt, über die die Patienten in vollem Umfang aufgeklärt werden. Falls nötig, werden die Patienten in Kooperation mit der Psychotherapie-Ambulanz und vorhandenen Kontakten dabei unterstützt, eine geeignete psychologische/psychiatrische Anbindung zu finden.

**Teilnahmebeschränkung.**  Die Teilnahmebeschränkungen beziehen sich auf die o.g. Ein- und Ausschlusskriterien. Darüber hinaus gibt es keine weiteren Einschränkungen.

**9. Informiertheit und Einwilligung**

**Informiertheit.** Das Prinzip der vollständigen Informiertheit ist zu allen Zeitpunkten der Erhebung gewahrt. Die Teilnehmer werden vollständig über den Zweck der Untersuchung informiert. Sie werden ausführlich über das Behandlungsprogramm, die Inhalte und Interventionen informiert.

**Einwilligung.** Nach Information der Teilnehmer wird die informierte Einwilligung eingeholt. In der Einwilligungserklärung erklären die Teilnehmer, ausführlich über die Untersuchung informiert worden zu sein, die Gelegenheit gehabt zu haben, Fragen zu stellen und zu verstehen, was die Untersuchung beinhaltet. Auch die Rücktrittsmöglichkeit ohne Nachteile für die Teilnehmer wird in der Einwilligungserklärung noch einmal wiederholt. Die Einwilligung enthält alle notwendigen Bestandteile (Freiwilligkeit, Informiertheit, volles Verständnis, Rücktrittsmöglichkeit ohne Nachteile).

**Bild- und Tonaufnahmen.** Von allen Sitzungen werden Videoaufnahmen erstellt. Diese Aufnahmen dienen ausschließlich dem Zweck der Qualitätssicherung der durchgeführten Intervention in der vorliegenden Studie. Aufgezeichnete Videos werden auf einer passwort-geschützten Festplatte gespeichert. Nur die Versuchsleitung und die Mitarbeiter kennen das Passwort zu der Festplatte. Ohne Passwort ist kein Zugang zu den Daten möglich. Die Teilnehmer werden über die Videoaufzeichnung aufgeklärt und erklären ihr Einverständnis (Einwilligung in Ton- und Bildaufnahmen) auf einem separaten Dokument. Nach Beendigung der Studie werden alle Videos gelöscht.

**Literaturangaben**

Baer, L. (1993). *Alles unter Kontrolle: Zwangsgedanken und Zwangshandlungen überwinden*. Bern: Hans Huber.

Belloch, A., Cabedo, E., Carrio, C. & Larsson, C. (2010).Cognitive therapy for autogenous and reactive obsessions: clinical and cognitive outcomes at post-treatment and 1-year follow-up. Journal of Anxiety Disorders, 24, 573–580.

Delespaul, P.A.E.G., Vries, de M.W., & Maastricht University. (1995, Mai 4). Assessing schizophrenia indaily life: the experience sampling method. UPM, Universitaire Pers Maastricht. Abgerufen von http://pub.maastrichtuniversity.nl/3b3255e1-8ee8-4675-8c3b-9f093afb355d

Ebner-Priemer, U. W. & Trull, T. J. (2009). Ecological momentary assessment of mood disorders and mood dysregulation. Psychological assessment, 21(4), 463.

Engel, S. G., Wonderlich, S. A., Crosby, R. D., Mitchell, J. E., Crow, S., Peterson, C. B., … Gordon, K. H. (2013). The role of affect in the maintenance of anorexia nervosa: Evidence from a naturalistic assessment of momentary behaviors and emotion. *Journal of Abnormal Psychology, 122*(3), 709–719.

Ertle, A., Wahl, K., Bohne, A., Moritz, S., Kordon, A. & Schulte, D. (2008). Dimensionen zwangsspezifischer Einstellungen: Der Obsessive-Beliefs Questionnaire (OBQ) für den deutschen Sprachraum analysiert. *Zeitschrift für Klinische Psychologie und Psychotherapie, 37,* 263-271.

Goldschmidt, A. B., Wonderlich, S. A., Crosby, R. D., Engel, S. G., Lavender, J. M., Peterson, C. B., …Mitchell, J. E. (2014). Ecological momentary assessment of stressful events and negative affect in bulimia nervosa. *Journal of Consulting and Clinical Psychology, 82*(1), 30–39.

Gönner, S., Leonhart, R., & Ecker, W. (2007). Das Zwangsinventar OCI-R – die deutsche Version des Obsessive-Compulsive Inventory-Revised. *Psychotherapie Psychosomatik Medizinische Psychologie, 57,* 395-404.

Hautzinger, M., Keller, F. & Kühner, C. (2006). *BDI II. Beck Depressions Inventar. Revision.* Frankfurt/Main: Harcourt Test Service.

Hand, I. & Büttner-Westphal H. (1991). Die Yale-Brown Obsessive Compulsive Scale (Y-BOCS): Ein halbstrukturiertes Interview zur Beurteilung des Schweregrades von Denk- und Handlungszwängen, *Verhaltenstherapie, 1,* 223-225.

Johansson, P. & Høglend, P. (2007). Identifying mechanisms of change in psychotherapy: Mediators of treatment outcome. Clinical Psychology and Psychotherapy, 14, 1–9.

Kordon, A., Lotz-Rambaldi, W., Muche-Borowski, C. & Hohagen, F. (2013). S3-Leitlinie Zwangsstörungen. Abgerufen von: http://www.awmf.org/uploads/tx_szleitlinien/038_017l_S3_Zwangsst%C3%B6rungen_2013.pdf

Kramer, I., Simons, C. J. P., Wigman, J. T. W., Collip, D., Jacobs, N., Derom, C., … Wichers, M. (2014). Time-Lagged Moment-to-Moment Interplay Between Negative Affect and Paranoia: New Insights in the Affective Pathway to Psychosis. *Schizophrenia Bulletin, 40*(2), 278–286.

Krueger, R. F., Derringer, J., Markon, K. E., Watson, D. & Skodol, A. E. (2012). Initial construction of a maladaptive personality trait model and inventory for DSM-5. *Psychological medicine, 42*(09), 1879-1890.

Lehrl, S. (2005). *Mehrfachwahl-Wortschatz-Intelligenztest.* Göttingen: Hogrefe.

Ludvik, D. & Boschen, M.J. (2015). Cognitive restructuring and detached mindfulness: Comparative impact on a compulsive checking task. Journal of Obsessive-Compulsive and Related Disorders, 5, 8-15.

Mills, J., Fuller-Tyszkiewicz, M. & Holmes, M. (2014). State Body Dissatisfaction and Social Interactions: An Experience Sampling Study. Psychology of Women Quarterly, 38(4), 551–562.

Myers, S.G., Fisher, P.L. & Wells, A. (2009). An empirical test of the metacognitive model of obsessive-compulsive symptoms: Fusion beliefs, beliefs about rituals, and stop signals. Journal of Anxiety Disorders, 23, 436-442.

Myers, S. G., & Wells, A. (2005). Obsessive-compulsive symptoms: The contribution of metacognitions and responsibility. *Journal of Anxiety Disorders*, *19*(7), 806-817.

Olatunji, B.O., Rosenfield, D., Tart, C.D., Cottraux, J., Powers, M.B. & Smits, J.A.J. (2013). Behavioral versus cognitive treatment of obsessive–compulsive disorder: an examination of outcome and mediators of change. Journal of Consulting and Clinical Psychology, 81, 415–428.

Palmier-Claus, J. E., Myin-Germeys, I., Barkus, E., Bentley, L., Udachina, A., Delespaul, P. a. E. G., … Dunn, G. (2011). Experience sampling research in individuals with mental illness: reflections and guidance. *Acta Psychiatrica Scandinavica, 123*(1), 12–20.

Purdon, C., Rowa, K. & Antony, M. M. (2007). Diary records of thought suppression by individuals with obsessive-compulsive disorder. Behavioural and Cognitive Psychotherapy, 35(01), 47-59.

Rosa-Alcázar, A.I., Sánchez-Meca, J., Gómez-Conesa, A. & Marín-Martínez., F. (2008). Psychological treatment of obsessive–compulsive disorder: a meta-analysis. Clinical Psychology Review, 28, 1310–1325.

Santangelo, P., Mussgay, L., Sawitzki, G., Trull, T. J., Reinhard, I., Steil, R., … Ebner-Priemer, U. W. (2014). Specificity of Affective Instability in Patients With Borderline Personality Disorder Compared to Posttraumatic Stress Disorder, Bulimia Nervosa, and Healthy Controls. *Journal of Abnormal Psychology, 123*(1), 258–272.

Schruers, K., Koning, K., Luermans, J., Haack, M.J. & Griez, E. (2005). Obsessive–compulsive disorder: a critical review of therapeutic perspectives. *Acta Psychiatrica Scandinavica, 111*, 261–271.

Schulte, D. (2005). Messung der Therapieerwartung und Therapieevaluation von Patienten (PATHEV). *Zeitschrift für Klinische Psychologie und Psychotherapie*, *34*(3), 176-187.

Shareh, H., Garraee, B., Atef-Vahis, M.K. & Eftekhar, M. (2010). Metacognitive Therapy (MCT), Fluvo-xamine, and Combinded Treatment in Improving Obsessive-Compulsive, Depressive and Anxiety Symptoms in Patients with Obsessive-Compulsive Disorder (OCD). Iranian Journal of Psychiatry and Behavioral Sciences, 4(2), 17-25.

Trull, T. J. & Ebner-Priemer, U. (2013). Ambulatory Assessment. *Annual Review of Clinical Psychology, 9*(1), 151–176.

Wells, A. (2011). *Metacognitive therapy for anxiety and depression*. Guilford press.

Wells, A., Gwilliam, P. & Cartwright-Hatton, S. (2002). Thought-Fusion-Instrument (TFI, Unveröffentlichtes Manuskript). University of Manchester, UK.

Wells, A. & McNicol, K. (2004). Beliefs About Rituals Inventory (BARI, Unveröffentlichtes Manuskript). University of Manchester, UK.

Wilhelm, S., Steketee, G., Fama, J.M., Buhlmann, U., Teachman, B.A. & Golan, E. (2009). Modular cognitive therapy for obsessive–compulsive disorder: a wait-list controlled trial. Journal of Cognitive Psychotherapy, 23, 294–305.

Wittchen, H. U., Zaudig, M. & Fydrich, T. (1997). *SKID. Strukturiertes Klinisches Interview für DSM-IV.* Göttingen: Hogrefe.
