# Supplementary material for: A randomized waitlist-controlled trial comparing detached mindfulness and cognitive restructuring in obsessive-compulsive disorder
Source: PLoS One. 2019 Mar 20;14(3):e0213895. doi: 10.1371/journal.pone.0213895 (PMC6426247; doi:10.1371/journal.pone.0213895)
Supplement: S6 File — This document includes the English translation of the original German study protocol that was approved of by the ethics committee. (DOCX) [file pone.0213895.s006.docx]

­­Request for Statement of the ethics committee of the Department of Psychology and Sport Science at the University of Münster, Germany

**1. Name of research project:** Dealing with intrusive thoughts in OCD – a comparison of two treatment strategies.

**2.** **Corresponding author, Name and contact address:**

Prof. Dr. Ulrike Buhlmann

Westfälische Wilhelms-University Münster

Institute of Psychology

Fliednerstrasse 21

48149 Münster

Germany

Phone: +49-251-8334112 | Fax: +49-251-8331331 | Email: [ulrike.buhlmann@wwu.de](mailto:rupp@cds-muenster.de)

**3. Framework information**

A statement of the ethics committee is required due to the planned publication of this research project and journal guidelines regarding a review and approval of an ethics committee.

We plan a randomized study with various assessment points. Psychometric data of persons suffering from obsessive-compulsive disorder (OCD) shall be assessed. Participation is voluntary and lasts 3 to 5 weeks (+ follow-up-assessment after 1 month). The study should be implemented in cooperation with the Christoph-Dornier-Foundation, Münster. This study shall be funded by Christoph-Dornier-Foundation.

**4. Subject and method of the planned project**

**Subject.** Concerning treatment guidelines (Kordon, Lotz-Rambaldi, Muche-Borowski & Hohagen, 2013, S.37) cognitive behavioral therapy containing exposure and response prevention as well as psychopharmacological treatment is considered as gold standard to treat OCD. Nevertheless, around 30% of the patients treated with cognitive behavioral therapy remain unimproved (Schruers, Koning, Luermans, Haack & Griez, 2005), thus, it seems to be crucial to further develop current treatments to improve therapy outcome.

The efficacy of cognitive therapy (CT) in the treatment of OCD has been shown in a number of trials (Rosa-Alcázar, Sánchez-Meca, Gómez-Conesa & Marin-Martínez, 2008; Wilhelm et al., 2009; Belloch, Cabedo, Carrio & Larrsson, 2010; Olatunji et al., 2013). Apart from that, metacognitive therapy (MCT) established by Wells (2011) represents another treatment choice. MCT focuses on changing one’s relationship to one’s own thoughts. Efficacy of complex MCT treatment programs for OCD has been proven in a number of trials (e.g., Shareh, Gharraee, Atef-Vahid & Eftekhar, 2010), however, applied treatments are composed of several stand-alone techniques. Therefore, no conclusion concerning efficacy of stand-alone techniques can be drawn. Research regarding the efficacy of single components of treatment programs seems to be crucial to improve the efficacy of treatments (see Johansson & Høglend, 2007).

According to Wells (2011), detached mindfulness (DM) is a key technique in MCT. It is a technique aiming at switching from “object mode“ to “metacognitive mode” and changing patient’s relation to their thoughts. To date, there are only two studies suggesting an efficacy of DM as stand-alone technique in reducing OCD symptoms (e.g., Ludvik & Boschen, 2015). Due to the design of these studies (individual case study, no clinical population), only limited conclusions can be drawn from these studies regarding the efficacy of DM.

Besides, it’s still unclear, how working mechanisms differ between cognitive and metacognitive interventions and whether cognitive or metacognitive beliefs can be regarded as relevant concerning the development and maintenance of OCD. Two studies (e.g, Myers & Wells, 2005) suggest that interventions, aiming at changing metacognitive beliefs might be faster effective than cognitive interventions.

In order to collect ecologically valid data (see Ebner-Priemer & Trull, 2009) regarding the course of a symptomatology Ecological Momentary Assessment (EMA) can be used. To our knowledge, EMA has been used in the research of OCD just twice to date (e.g., Purdon, Rowa & Anthony, 2007). EMA-data regarding cognitive and metacognitive changes during interventions have not been presented.

This study aims at answering the following research questions:

1. Does the constant use of DM leads to a significant reduction of OCD symptoms (compared to a waiting-list control group)?
2. Does the constant use of cognitive restructuring leads to a significant reduction of OCD symptoms (compared to a waiting-list control group)?
3. How easily to patients succeed in the application of the particular method?
4. Is there a significant difference between DM and cognitive restructuring regarding the reduction of the frequency and intensity of OCD symptoms?
5. Does the psychoeducation leads to a significant reduction of OCD symptoms?
6. Is there a difference between the different forms of psychoeducation (MCT/CT) regarding its effect?
7. How do OCD symptoms (frequency of obsessions, burden due to obsessions, emotions related to obsessions, strength of urge to respond to obsessions; behavior in reply to obsessions) occur in the patient’s everyday life?
8. How does the dealing with obsessions changes in the course of the interventions (frequency of application of the strategy; difficulties of application, relief due to the new strategy) (measured by questionnaires and EMA)?

**Methods.** Data collection is based on self-report questionnaires which participants complete on a tablet before each session using a web-based online survey-software (*Unipark*). Diagnostic assessments at the beginning (session I & Ib) and at the end of the study (session VI & VII) comprise a clinical interview to assess mental disorders as well as an interview to assess the severity of OCD. Besides, a verbal intelligence test is applied during the Pre1 assessment. Diagnostic assessments as well the implementation of EMA are conducted by an independent clinical evaluator. All evaluators are Master level psychologists currently participating in an advanced training to become a cognitive behavioral psychotherapist.

These questionnaires will be used:

| **measure** | **description** | **session Ia** | **session Ib** | **session II** | **session III** | **session IV** | **session V** | **session VI** | **session VII** |
| --- | --- | --- | --- | --- | --- | --- | --- | --- | --- |
| **Anamnesis form** | Date of birth, gender, citizenship, family status, children, highest general education certificate, age of receiving highest general education certificate, years of school education, highest professional qualification, age of receiving highest professional qualification, duration of post-school professional qualification, employment, OCD age of onset, age of onset being burdened by OCD, Suicidality (current and in the past), current psychiatric treatment, current psychiatric medication, psychotherapeutic treatment in the past, number of previous inpatient & outpatient psychotherapeutic treatments, forms of psychotherapeutic treatment, date of last psychotherapeutic session | √ | ---- | ---- | ---- | ---- | ---- | ---- | ---- |
| **Structured Clinical Interview for DSM-IV Axis I Disorders** (SCID-I; Wittchen, Zaudig, & Fydrich, 1997) | Structured Clinical Interview to assess mental disorders according to DSM-IV | √ | (only *OCD section*) | ---- | ---- | ---- | ---- | (only *OCD section*) | (only *OCD section*) |
| German version of **Y-BOCS** (Hand & Büttner-Westphal, 1991) | Semi-structured interview to assess the severity of obsessions and compulsions regarding the last two weeks. | √ | √ | ---- | ---- | ---- | ---- | √ | √ |
| German equivalent of the **self-rating version of the Y-BOCS** (Baer, 1993) | Self-rating scale to assess the severity of obsessions and compulsions | √ | √ | ---- | √ | √ | √ | √ | √ |

**√** = application in session / **----**  = no application in session

| German version of **Beck Depression Inventory-Revised** (BDI-II, Hautzinger, Keller, & Kühner, 2006) | 21-item self-rating questionnaire to assess the severity of depressive symptoms in the last two weeks | √ | √ | ---- | √ | √ | √ | √ | √ |
| --- | --- | --- | --- | --- | --- | --- | --- | --- | --- |
| **Multiple-choice vocabulary intelligence test** (MWT, Lehrl, 2015). | Verbal achievement test to assess general intelligence | √ | ---- | ---- | ---- | ---- | ---- | ---- | ---- |
| German version (Gönner, Leonhart, & Ecker, 2007) of the **Obsessive-Compulsive Inventory-Revised** (Foa et al., 2002) | 18-item self-rating scale to assess the severity, main symptoms and symptom dimensions of OCD on 6 subscales (washing, checking, ordering, obsessions, hoarding, mental neutralizing) | √ | √ | ---- | √ | √ | √ | √ | √ |
| German translation of the **Thought-Fusion Instrument** (Wells, Gwilliam, & Cartwright-Hatton, 2002) | 14-item self-report scale assessing beliefs about the meaning and power of thoughts on 3 subscales (thought action fusion, thought event fusion, thought object fusion) | √ | √ | ---- | √ | √ | √ | √ | √ |
| German translation of **Beliefs About Rituals Inventory** (Wells & McNicol, 2004), | 12-item self-report scale measuring positive beliefs about rituals | √ | √ | ---- | √ | √ | √ | √ | √ |
| German translation of **Stop Signals Questionnaire** (Myers, Fisher, & Wells, 2009) | 12-item self-report scale to rate the relevance of various signals in deciding when to stop OC-rituals | √ | √ | ---- | √ | √ | √ | √ | √ |
| German version (Ertle et al., 2008) of the **Obsessive-Beliefs Questionnaire** (Obsessive Compulsive Cognitions Working Group, 2005) | 24-items self-report scale to assess attitudes towards and beliefs about intrusive thoughts on 3 subscales (meaning of thoughts/ necessity to control thoughts, perfectionism/intolerance of uncertainty, overassessment of danger/ inflated responsibility) | √ | √ | ---- | √ | √ | √ | √ | √ |
| The German version of **Personality Inventory for DSM-5** (American Psychiatric Association, 2015) | 100-items self-report scale to assess personality traits | √ | ---- | ---- | ---- | ---- | ---- | ---- | ---- |
| **Homework Rating** | Self-rating scale to assess feasibility and effectiveness of the strategy learned in session | ---- | ---- | ---- | ---- | ---- | √ | √ | √ |
| German version of **Patient Questionnaire on Therapy Expectation and Evaluation,** (PATHEV; Schulte, 2005) | 11-items self-rating scale to assess the expectations and evaluation of the implemented therapy | ---- | ---- | ---- | ---- | ---- | ---- | √ | √ |

On two dates, EMA-data are assessed (after session II and VI). Participants receive a smartphone with touchscreen for a period of 4 days (Friday to Monday) which is programmed in a way that participants can only use it for data collection within the EMA study. At 10 time points a day (these are randomly chosen, with a distance of at least 30 minutes between the time points) the smartphone makes a sound signal indicating a short survey that lasts around 2 min.

Within 15 min after the sound signal, the participant can answer the survey on the smartphone. In case he/she does not react in this period of time, during which the sound signal appears up to 5 times to indicate the survey, this survey time point drops out. This time limit is based on considerable data by Delespaul (1995), indicating that 15 min after a signal, the reliability of answers decreases, so that these answers should not be included in the data analysis. Every time a signal appears, participants have the possibility of delaying a survey to a later time point or to skip the time point completely by pushing the relevant buttons. Besides, they have the possibility of muting the smartphone for the duration of one hour by pushing the relevant button (“Do not disturb”). In this case, no sound signals appear during this period of time.

The following aspects are assessed during the EMA-assessment on 7-point Likert-scales:

After session II (Pre-EMA):

- Level of stress since the last signal
- Level of relaxation since the last signal
- Frequency of obsessions since the last signal
- Burden due to obsessions since the last signal
- Emotions related to the obsessions
- Frequency of specific responses to obsessions since the last signal

After session VI (Post-EMA): the same items as in the Pre-EMA survey are presented, in and addition:

- Frequency of the application of the newly learned technique to deal with obsessions since the last signal
- Subjective difficulties during the application of the newly learned technique since the last signal
- Subjective relief by the application of the newly learned technique since the last signal

Moreover, in both EMA-assessments, each participant is asked if persons (known/unknown) have been nearby since the last signal.

A pilot study examining 3 mentally healthy participants and 83 answered signals found a mean processing time of 1:28 min, so that an estimation of the processing time per answered signal of around 2 min seems realistic.

**Experimental tasks.** None.

**Implementation.** Participants are recruited via the psychotherapeutic outpatient department of the University of Münster (head: Prof. Ulrike Buhlmann) as well as via the Christoph-Dornier-Foundation (head: Dr. Fabian Andor). Further participants should be recruited via posters, flyers, advertisements and postings.

Afterwards, a phone screening is conducted. Participants meeting the inclusion criteria then are invited to an assessment session. The subsequent sessions are conducted on seven appointments (respectively eight appointments in the waiting list group). Session VI comprises the last therapeutic session as well as the post-assessment, which is conducted directly after the last therapeutic session but is not conducted by the therapist but the same independent clinical evaluator as in Pre-assessment in session Ia (and possibly in Ib).

Each treatment session (sessions III to VI) lasts 100 min. Regarding session VI, participants should plan around 180 min as the Post-assessment is conducted afterwards. Session Ia lasts around 180 min too, session Ib and session VII last around 90 min, which can be regarded as a generous estimation. The session to implement the EMA-assessment takes around 45 min as the detailed explanation of the items, which are adjusted by the evaluator to the problem area of each participant, take time. At the beginning of each session, participants are asked to complete the questionnaires mentioned above. To avoid mistakes, completion of the questionnaires is computer-based on a tablet (via *Unipark*). Content of each session is described in the following overview:

**Schematic description of the procedure:**

**SESSION I (diagnostic session; conducted by diagnostic evaluator):**

- Handout of participant information form and filling in the declaration of consent
- Collection of demographic data using an participant history questionnaire
- Completion of SKID-I, Y-BOCS and MWT
- Completion of the questionnaires
- In parallel: Screening of criteria for inclusion and exclusion

**Randomized group assignment and disclosure of the assignment via phone call:** participants of the waiting list control group (WL) pause for two weeks; following this, pre-diagnostics are conducted once more (*session Ib*)

**SESSION Ib (WL only; conducted by diagnostic evaluator):**

- Completion of the section „Obsessive-Compulsive Disorder“ of the SCID-I and Y-BOCS
- Completion of the questionnaires

**SESSION II (EMA-introduction; conducted by diagnostic evaluator):**

- Introduction to the use of the EMA-smartphones

**SESSION III (conducted by therapist):**

- Completion of the questionnaires
- Psychoeducation on OCD
- Development of a cognitive or meta-cognitive maintenance model of OCD (depending on experimental condition)
- Depending on experimental condition: Deduction of cognitive restructuring (CT) strategies or strategies of DM

**SESSION IV (conducted by therapist):**

- Completion of the questionnaires
- Introduction of CT/ DM
- Practice of CT/ DM

**SESSION V (conducted by therapist):**

- Completion of the questionnaires
- Practice of CT/ DM

**SESSION VI (conducted by therapist & diagnostician):**

- Completion of the questionnaires
- Résumé on the efficacy of the learned strategy
- Development of an effective relapse prophylaxis
- Completion of Y-BOCS and OCD the SCID-I
- Completion of the PATHEV
- Explanation of the three new EMA-items and handing out of the smartphone

**SESSION VII (conducted by diagnostic evaluator)**

- Completion of Y-BOCS and OCD section of SCID-I
- Completion of the questionnaires
- Participants receive the allowance

**Physical load.** None.

**Mental load.** Mental load of the participants is medium and results mainly from the requirement of concentration when participating in diagnostics and therapy sessions.

Furthermore, the participants answer questions concerning negative life domains. In the course of diagnostic assessment the focus is on absorbing these strains and accurately assessing the eligibility for participating in the study. For this reason, all contacts with the participants are conducted by trained psychotherapists, which are in an advanced stage of their therapist education and are available for phone calls when the participants experience strains. If they are not available, the participants can leave a message on the voice mail and are called back as soon as possible within the working hours. In addition, the participants can contact the examiners via an e-mail address given in the participant information handout. This e-mail address is checked on workdays within 48 hours, and questions are answered as soon as possible. In the case of acute self-endangerment or endangerment of others an internal emergency action plan of the psychotherapy outpatient department of the University of Münster is available. All employees are familiar with the respective procedures and initiate the necessary steps in case of emergency or contact the examiner immediately.

Because of the smartphone-based assessment, an additional load is not to be expected according to the reviews concerning EMA by Palmier-Claus et al. (2011) and Trull and Ebner-Priemer (2013) even in participants with severe mental disorders such as schizophrenia, depression and Borderline personality disorder.

**Disclosure of personal information**. We collect sociodemographic variables, information on mental disorders and treatments and questionnaire dimensions about obsessive and compulsive symptoms. The collection of this information is indispensable for the study. The information cannot be traced back to the participants after the collection stage of the study due to a pseudonymized procedure.

**Deceit and clarification.** Before giving their informed consent, the participants receive complete information on the purpose of the investigation and the procedure of the study, i.e. the participants are not deceived.

**5. Information on recording, processing and deleting the data**

**Personal data.** Sociodemographic data are collected from all participants. The names of the participants are used only for the documentation of informed consent as well as for the coding list (see below).

**Privacy.** Pursuant § 3 passage 1 of the Federal Data Protection Act, as few personal data as possible are collected, processed and saved. The diagnostic data are pseudonymized, i.e. saved without mentioning participants by name but using a participant code. There is a written coding list on paper, which matches the name with the respective participant code. This coding list is only accessible to the examiners, which are liable to professional secrecy. Each student/employee involved in the study signs a statement of professional secrecy for this reason.

The computer-based assessment is conducted without the collection of personal data via *Unipark*, a software for online surveys by *Questback*.

On the smartphone, the data saved so far can only be accessed with an administrator password which is only known by the responsible employee. The pseudonymized data on the smartphone cannot be traced back to the participant without the coding list. Should a smartphone of a participant get lost or stolen, it can additionally be reset to the factory settings via the Android Device Manager, such that all data on the smartphone can be deleted.

**Coding list.** The coding list is kept in a locker and destroyed after completion of data processing, but soonest after one year. After destruction of the coding list the anonymized data will be saved for at least 10 years (they cannot be traced back to the individual participants anymore). The participants are asked whether they agree with being contacted again for further scientific investigations and can state in the declaration of informed consent that they disagree without having to give reasons for that.

**Deletion of the data.** As long as the coding list exists, participants can always claim the deletion of all data collected from them. The coding list is destroyed after completion of data processing, but soonest after one year.

**6. Recruitment and compensation of participants**

**Recruitment.** Recruitment of the sample will be done via the OCD section of the psychotherapeutic outpatient department of the University of Münster („Psychotherapie-Ambulanz“, responsible: Prof. Ulrike Buhlmann) and via the outpatient department of the Christoph-Dornier-Foundation Münster (responsible: Dr. Fabian Andor). Furthermore, patients shall be recruitment via postings and newspaper advertisements.

**Human sample out of database?** No database will be used for recruitment.

**Characteristics of the human sample.** We plan to recruit 60 persons currently suffering from a clinical level of OCD. Only persons at least 18 years of age shall be included in the study.

**Inclusion and exclusion criteria.** Inclusion criterion: Current diagnosis of clinically relevant OCD as primary diagnosis (based on current amount of symptom burden); if under medication: a dose that has been stable since eight weeks.

Exclusion criteria:

- A disorder other than OCD being the primary diagnosis
- Age < 18 years
- Not fluent in German
- Verbal IQ < 80 (since in that case one cannot assume that cognitive abilities required for treatment are given)
- Acute suicidality or suicidal behavior within the past six months
- Acute psychosis or validated diagnosis of a schizophrenic disease (ICD-10: F2), current or lifetime
- Acute manic episode
- Current substance dependency disorder
- Acute borderline personality disorder
- Current psychotherapeutic treatment involving OCD-specific CBT interventions or psychotherapeutic treatment involving OCD-specific CBT interventions within the past year
- Change of dose or substance concerning a psychiatric medication within the past eight months or during study participation.

**Web-based data collection.** Participants will fill in the questionnaires via the internet platform Unipark. While doing to, the evaluator or therapist will be approachable so that uncertainties and potential problems concerning the touch screen-based data collection can be solved in case of participants who are not familiar with the technology.

**Attendance allowance**. Participants will be paid € 30 each for participation in Pre and Post assessment, and participation in FU assessment will be allowed with € 40 in order to reduce the risk of dropout prior to FU. Participants who are initially allocated to the waitlist condition receive an additional € 40 for the second Pre assessment.

The allowance is paid following FU assessment. In case of dropout, participants are paid an allowance that corresponds to the amount of time they have invested until then for the purpose of assessment sessions. The participants sign that they have received the allowance.

Filling in the EMA items will be additionally allowed €40 per sampling period. In case participants respond to at least 80% of all smartphone prompts they will be allowed a bonus allowance of €20. We assume that receiving the bonus is equally achievable for all participants regardless of demographic or psychopathological characteristics. In order to obtain a realistic impression concerning the course of the variables of interest, we will not make any individual adjustments concerning this. Prior to the smartphone-based sampling period we will explore individual potentially difficult situations (such as the handling of the smartphone at work) and weigh possible ways of dealing with it (e. g., tell trusted people about study participation, leave out certain prompts). The influence of a potential disadvantage for people working full-time concerning the bonus is reduced markedly by the fact that 50% of the sampling takes place on weekend days, i. e. on Saturdays and Sundays.

In published EMA studies using similar designs with adult participants average compliance is very high (cf. Review of Trull & Ebner-Priemer, 2013), so that we assume that a compliance rate of 80% can be achieved in the study at hand even in spite of employment and shift duty. Furthermore, such a bonus is common in daily practice in order to increase compliance rates, see e. g. Engel et al. (2013) who paid a € 50 bonus at 80% compliance.

Examples of compliance rates:

- Engel et al. (2013): average compliance of 87% among n = 118 participants with clinical and subclinical anorexia. According to the analyses conducted, the compliance rate was not influenced by demographic characteristics except age
- Goldschmidt et al. (2014): average compliance of 86% and 75% within 20 minutes after the signal among n=133 participants with bulimia
- Kramer et al. (2014): average compliance of 74% among n=515 women from a sample of twins (60% working)
- Mills et al. (2014) average compliance of 84% in a sample of n=128 women (39% working full-time, 36% working part-time)
- Santangelo et al. (2014): average compliance of 94% among n=43 participants with Borderline disorder, n=28 with PTSD, n=20 with bulimia und 28 mentally healthy controls.

**7. Voluntariness of participation and withdrawal.**

**Voluntariness.** Measures for ensuring voluntariness are given. During the first appointment, participants receive a clarification sheet about the background of the study in which they are explicitly made aware of the voluntariness of their participation. They are given enough time to decide for or against participation. Participants are made explicitly told that they cannot expect any advantages due to study participation and that there will be no disadvantages in case of not participating.

**Withdrawal.** Participants are explicitly told that they can withdraw from the study at any time without facing disadvantages. Participants are explicitly told that the collection of questionnaire data is done in a pseudonymized, form i. e., without including their names. They are aware of the fact that until the end of data collection there exists a coding list that can be accessed by the investigators and that will be destroyed after completion of data collection. Participants know that they can demand deletion of their data as long as the coding list exists and that this will be the case or a minimum of one year. Participants are told that in case of withdrawal they can be put on the regular waitlist for CBT treatment in the Christoph-Dornier-Foundation or the psychotherapeutic outpatient department of the University of Münster if they seek psychotherapeutic treatment.

**8. Dealing with incidental findings**

**Enlightenment.** Bodily incidental findings are not to be expected in this study. In the course of diagnostic assessment psychological disorder will be diagnosed, and patients will be fully enlightened about the diagnoses. If necessary, patients will be supported in finding adequate psychological/psychiatric treatment in cooperation with the psychotherapeutic outpatient department.

**Restriction concerning participation.** Restrictions concerning participation refer to the inclusion and exclusion criteria. Apart from that there are no further restrictions.

**9. Knowledgeability and consent**

**Knowledgeability.** The principle of full knowledgeability is ensured at all points of the study. Participants are fully informed about the purpose of the study. They are informed in detail about the treatment program, its contents and interventions.

**Consent.** After information, the participants are asked to provide their informed consent. In the consent form the participants declare that have been informed about the study in detail, have had the opportunity to ask questions and to understand what the study entails. The consent form also includes once more the possibility of withdrawing/declining without disadvantage. The consent includes all necessary elements (voluntariness, knowledgeability, full understanding, possibility of withdrawing without disadvantages).

**Video and audio recordings.** There will be video and audio recordings of all sessions. These recordings fulfill the single purpose of monitoring the quality of the interventions delivered within this study. The videos are saved on a password-protected hard drive. Only the investigators and the assistants know the password of the hard drive. The participants are informed about the video recordings and declare their consent (consent concerning video and audio recordings) on a separate sheet. After completion of the study all videos are deleted.

**References**

Baer, L. (1993). *Alles unter Kontrolle: Zwangsgedanken und Zwangshandlungen überwinden*. Bern: Hans Huber.

Belloch, A., Cabedo, E., Carrio, C. & Larsson, C. (2010).Cognitive therapy for autogenous and reactive obsessions: clinical and cognitive outcomes at post-treatment and 1-year follow-up. Journal of Anxiety Disorders, 24, 573–580.

Delespaul, P.A.E.G., Vries, de M.W., & Maastricht University. (1995, Mai 4). Assessing schizophrenia indaily life: the experience sampling method. UPM, Universitaire Pers Maastricht. Abgerufen von http://pub.maastrichtuniversity.nl/3b3255e1-8ee8-4675-8c3b-9f093afb355d

Ebner-Priemer, U. W. & Trull, T. J. (2009). Ecological momentary assessment of mood disorders and mood dysregulation. Psychological assessment, 21(4), 463.

Engel, S. G., Wonderlich, S. A., Crosby, R. D., Mitchell, J. E., Crow, S., Peterson, C. B., … Gordon, K. H. (2013). The role of affect in the maintenance of anorexia nervosa: Evidence from a naturalistic assessment of momentary behaviors and emotion. *Journal of Abnormal Psychology, 122*(3), 709–719.

Ertle, A., Wahl, K., Bohne, A., Moritz, S., Kordon, A. & Schulte, D. (2008). Dimensionen zwangsspezifischer Einstellungen: Der Obsessive-Beliefs Questionnaire (OBQ) für den deutschen Sprachraum analysiert. *Zeitschrift für Klinische Psychologie und Psychotherapie, 37,* 263-271.

Goldschmidt, A. B., Wonderlich, S. A., Crosby, R. D., Engel, S. G., Lavender, J. M., Peterson, C. B., …Mitchell, J. E. (2014). Ecological momentary assessment of stressful events and negative affect in bulimia nervosa. *Journal of Consulting and Clinical Psychology, 82*(1), 30–39.

Gönner, S., Leonhart, R., & Ecker, W. (2007). Das Zwangsinventar OCI-R – die deutsche Version des Obsessive-Compulsive Inventory-Revised. *Psychotherapie Psychosomatik Medizinische Psychologie, 57,* 395-404.

Hautzinger, M., Keller, F. & Kühner, C. (2006). *BDI II. Beck Depressions Inventar. Revision.* Frankfurt/Main: Harcourt Test Service.

Hand, I. & Büttner-Westphal H. (1991). Die Yale-Brown Obsessive Compulsive Scale (Y-BOCS): Ein halbstrukturiertes Interview zur Beurteilung des Schweregrades von Denk- und Handlungszwängen, *Verhaltenstherapie, 1,* 223-225.

Johansson, P. & Høglend, P. (2007). Identifying mechanisms of change in psychotherapy: Mediators of treatment outcome. Clinical Psychology and Psychotherapy, 14, 1–9.

Kordon, A., Lotz-Rambaldi, W., Muche-Borowski, C. & Hohagen, F. (2013). S3-Leitlinie Zwangsstörungen. Abgerufen von: http://www.awmf.org/uploads/tx_szleitlinien/038_017l_S3_Zwangsst%C3%B6rungen_2013.pdf

Kramer, I., Simons, C. J. P., Wigman, J. T. W., Collip, D., Jacobs, N., Derom, C., … Wichers, M. (2014). Time-Lagged Moment-to-Moment Interplay Between Negative Affect and Paranoia: New Insights in the Affective Pathway to Psychosis. *Schizophrenia Bulletin, 40*(2), 278–286.

Krueger, R. F., Derringer, J., Markon, K. E., Watson, D. & Skodol, A. E. (2012). Initial construction of a maladaptive personality trait model and inventory for DSM-5. *Psychological medicine, 42*(09), 1879-1890.

Lehrl, S. (2005). *Mehrfachwahl-Wortschatz-Intelligenztest.* Göttingen: Hogrefe.

Ludvik, D. & Boschen, M.J. (2015). Cognitive restructuring and detached mindfulness: Comparative impact on a compulsive checking task. Journal of Obsessive-Compulsive and Related Disorders, 5, 8-15.

Mills, J., Fuller-Tyszkiewicz, M. & Holmes, M. (2014). State Body Dissatisfaction and Social Interactions: An Experience Sampling Study. Psychology of Women Quarterly, 38(4), 551–562.

Myers, S.G., Fisher, P.L. & Wells, A. (2009). An empirical test of the metacognitive model of obsessive-compulsive symptoms: Fusion beliefs, beliefs about rituals, and stop signals. Journal of Anxiety Disorders, 23, 436-442.

Myers, S. G., & Wells, A. (2005). Obsessive-compulsive symptoms: The contribution of metacognitions and responsibility. *Journal of Anxiety Disorders*, *19*(7), 806-817.

Olatunji, B.O., Rosenfield, D., Tart, C.D., Cottraux, J., Powers, M.B. & Smits, J.A.J. (2013). Behavioral versus cognitive treatment of obsessive–compulsive disorder: an examination of outcome and mediators of change. Journal of Consulting and Clinical Psychology, 81, 415–428.

Palmier-Claus, J. E., Myin-Germeys, I., Barkus, E., Bentley, L., Udachina, A., Delespaul, P. a. E. G., … Dunn, G. (2011). Experience sampling research in individuals with mental illness: reflections and guidance. *Acta Psychiatrica Scandinavica, 123*(1), 12–20.

Purdon, C., Rowa, K. & Antony, M. M. (2007). Diary records of thought suppression by individuals with obsessive-compulsive disorder. Behavioural and Cognitive Psychotherapy, 35(01), 47-59.

Rosa-Alcázar, A.I., Sánchez-Meca, J., Gómez-Conesa, A. & Marín-Martínez., F. (2008). Psychological treatment of obsessive–compulsive disorder: a meta-analysis. Clinical Psychology Review, 28, 1310–1325.

Santangelo, P., Mussgay, L., Sawitzki, G., Trull, T. J., Reinhard, I., Steil, R., … Ebner-Priemer, U. W. (2014). Specificity of Affective Instability in Patients With Borderline Personality Disorder Compared to Posttraumatic Stress Disorder, Bulimia Nervosa, and Healthy Controls. *Journal of Abnormal Psychology, 123*(1), 258–272.

Schruers, K., Koning, K., Luermans, J., Haack, M.J. & Griez, E. (2005). Obsessive–compulsive disorder: a critical review of therapeutic perspectives. *Acta Psychiatrica Scandinavica, 111*, 261–271.

Schulte, D. (2005). Messung der Therapieerwartung und Therapieevaluation von Patienten (PATHEV). *Zeitschrift für Klinische Psychologie und Psychotherapie*, *34*(3), 176-187.

Shareh, H., Garraee, B., Atef-Vahis, M.K. & Eftekhar, M. (2010). Metacognitive Therapy (MCT), Fluvo-xamine, and Combinded Treatment in Improving Obsessive-Compulsive, Depressive and Anxiety Symptoms in Patients with Obsessive-Compulsive Disorder (OCD). Iranian Journal of Psychiatry and Behavioral Sciences, 4(2), 17-25.

Trull, T. J. & Ebner-Priemer, U. (2013). Ambulatory Assessment. *Annual Review of Clinical Psychology, 9*(1), 151–176.

Wells, A. (2011). *Metacognitive therapy for anxiety and depression*. Guilford press.

Wells, A., Gwilliam, P. & Cartwright-Hatton, S. (2002). Thought-Fusion-Instrument (TFI, Unveröffentlichtes Manuskript). University of Manchester, UK.

Wells, A. & McNicol, K. (2004). Beliefs About Rituals Inventory (BARI, Unveröffentlichtes Manuskript). University of Manchester, UK.

Wilhelm, S., Steketee, G., Fama, J.M., Buhlmann, U., Teachman, B.A. & Golan, E. (2009). Modular cognitive therapy for obsessive–compulsive disorder: a wait-list controlled trial. Journal of Cognitive Psychotherapy, 23, 294–305.

Wittchen, H. U., Zaudig, M. & Fydrich, T. (1997). *SKID. Strukturiertes Klinisches Interview für DSM-IV.* Göttingen: Hogrefe.
